# Contrasting model mechanisms of alanine aminotransferase (ALT) release from damaged and necrotic hepatocytes as an example of general biomarker mechanisms

**DOI:** 10.1371/journal.pcbi.1007622

**Published:** 2020-06-02

**Authors:** Andrew K. Smith, Glen E. P. Ropella, Mitchell R. McGill, Preethi Krishnan, Lopamudra Dutta, Ryan C. Kennedy, Hartmut Jaeschke, C. Anthony Hunt

**Affiliations:** 1 Department of Bioengineering and Therapeutic Sciences, University of California, San Francisco, California, United States of America; 2 Tempus Dictum, Inc., Milwaukie, Oregon, United States of America; 3 Department of Environmental and Occupational Health, Fay W. Boozman College of Public Health, University of Arkansas for Medical Sciences, Little Rock, Arkansas, United States of America; 4 Department of Pharmacology, Toxicology and Therapeutics, University of Kansas Medical Center, Kansas City, Kansas, United States of America; US Army Medical Research and Materiel Command, UNITED STATES

## Abstract

Interpretations of elevated blood levels of alanine aminotransferase (ALT) for drug-induced liver injury often assume that the biomarker is released passively from dying cells. However, the mechanisms driving that release have not been explored experimentally. The usefulness of ALT and related biomarkers will improve by developing mechanism-based explanations of elevated levels that can be expanded and elaborated incrementally. We provide the means to challenge the ability of closely related model mechanisms to generate patterns of simulated hepatic injury and ALT release that scale (or not) to be quantitatively similar to the wet-lab validation targets, which are elevated plasma ALT values following acetaminophen (APAP) exposure in mice. We build on a published model mechanism that helps explain the generation of characteristic spatiotemporal features of APAP hepatotoxicity within hepatic lobules. Discrete event and agent-oriented software methods are most prominent. We instantiate and leverage a small constellation of concrete model mechanisms. Their details during execution help bring into focus ways in which particular sources of uncertainty become entangled with cause-effect details within and across several levels. We scale ALT amounts in virtual mice directly to target plasma ALT values in individual mice. A virtual experiment comprises a set of Monte Carlo simulations. We challenge the sufficiency of four potentially explanatory theories for ALT release. The first of the tested model theories failed to achieve the initial validation target, but each of the three others succeeded. Results for one of the three model mechanisms matched all target ALT values quantitatively. It explains how ALT externalization is the combined consequence of lobular-location-dependent drug-induced cellular damage and hepatocyte death. Falsification of one (or more) of the model mechanisms provides new knowledge and incrementally shrinks the constellation of model mechanisms. The modularity and biomimicry of our explanatory models enable seamless transition from mice to humans.

## Author summary

Interpretations of elevated biomarkers for drug-induced liver injury assume passive release during hepatocyte death, yet indirect evidence indicates that plasma levels can increase absent injury. Limitations on measurements make it infeasible to resolve causal linkages between drug disposition and plasma levels of biomarkers. To improve explanatory knowledge, we instantiate within virtual mice, plausible mechanism-based causal linkages between acetaminophen disposition and alanine aminotransferase (ALT) behavior that enables simulation results to meet stringent quantitative validation prerequisites. We challenge the sufficiency of four model mechanisms by scaling ALT amounts in virtual mice to corresponding plasma values. Virtual experiment results in which ALT externalization is a combined consequence of lobular-location-dependent hepatocyte death and drug-induced cellular damage, matches all validation targets. We assert that the actual mechanisms responsible for plasma ALT values in individual mice and the virtual causal processes occurring during model execution are strongly analogous within and among real hepatic lobular levels.

This is a *PLOS Computational Biology* Methods paper.

## Introduction

The use of several conventional clinical biomarkers (e.g., alanine aminotransferase (ALT), aspartate aminotransferase, creatine kinase, lipase, cardiac troponin, etc.) assumes that the analytes are passively released from dying cells [1,2]. However, the mechanisms have not been resolved experimentally. We seek improved understanding of the temporal events leading to and driving biomarker release to aid their interpretation, especially in cases where biomarker measures may be linked to medications, and to inform the identification, validation, and use of future biomarkers. We begin by focusing on ALT release from parenchymal cells (hepatocytes) in the liver.

ALT is a good candidate for developing plausible release mechanisms. It has been measured in circulation since the 1950s [3] and has become the standard clinical biomarker of liver injury. Because transient ALT elevations are frequently observed during preclinical testing and clinical trials of new drugs, the correct interpretation of its measures can be critical [2]. There may be considerable diversity in the processes linking ALT release with different initiators of tissue damage. To enable challenging competing ALT release hypotheses, we start by focusing on acetaminophen (APAP), a widely used, well-studied analgesic that, when overdosed, causes hepatotoxicity. It is generally assumed that elevated measures of serum ALT are a direct consequence of release from hepatocytes undergoing necrosis, yet some patients treated with therapeutic doses of APAP can experience transient elevations absent any evidence of liver injury [4]. Large variations in serum ALT values across preclinical models are another barrier to interpretation. For example, Harrill et al. reported a 9 to 20-fold variation in mean serum ALT among different mouse strains that received a standard toxic APAP dose [5].

The main objective of this work is to develop, support, and challenge plausible cause-effect linkages between APAP disposition and metabolism and concurrent measurements of ALT in plasma. Doing so is a requisite for resolving plausible explanations for variation within and among studies and thereby diminishing barriers to interpretation. However, it is currently infeasible to establish such linkages in vivo because hepatocyte damage and resulting ALT release cannot be measured directly. Despite these barriers and uncertainties, progress is being made using conventional mathematical modeling methods. For example, Howell et al. used a multifaceted drug-induced liver injury model to offer new insights into serum ALT values from several studies [6]. Their hypothetical ALT release sub-model assumes that ALT is released by necrotic cells. It describes released ALT moving to intralobular spaces and then into the blood.

The target wet-lab phenomena for this work are elevated plasma ALT values in six mice at 3, 4.5, and 6 hours following a toxic APAP dose [7]. Building on earlier work [8], we developed, implemented, and tested virtual counterparts to four hypotheses that identify the immediate cause of ALT externalization from individual hepatocytes into the circulation. The mechanism is a consequence of: 1) only hepatocyte death, 2) mitochondrial damage with or without later hepatocyte death, 3) general cell damage, other than mitochondrial, with or without later hepatocyte death, or 4) concurrent mitochondrial plus general cell damage with or without later hepatocyte death. In 2–4, we say “with or without” because we hypothesize that some hepatocytes recover from early mitochondrial and general cell damage, depending on their periportal (PP) to pericentral (PC) location within hepatic lobules, whereas necrosis is triggered in other hepatocytes before recovery has advanced sufficiently.

Smith et al. [8], provide a quantitative model mechanism that explains characteristic early spatiotemporal patterns APAP-induced hepatic necrosis. We extend that mechanism to include a counterpart of each of the four hypotheses. We scale ALT in virtual mouse bodies (excluding the liver) directly to target plasma ALT values. Based on results of virtual experiments, we reject the first hypothesis, as detailed here, because amounts of ALT in virtual mouse bodies could not be scaled to quantitatively match average target plasma ALT values. However, the results support Hypotheses 2–4. We limited detailed testing to the virtual counterpart of Hypothesis 2 because it is the most parsimonious of the remaining hypotheses. We discovered a parameterized version that enabled scaling amounts of ALT in virtual mouse bodies to quantitatively match target plasma ALT values from all 18 mice. Ours is the first cause-effect model mechanism to provide plausible explanations for entanglements of APAP metabolism and hepatic disposition, accumulation of toxic damage, externalization of ALT from hepatocytes, and ALT accumulation in plasma following a toxic APAP dose in mice.

Over time, knowledge within these model mechanisms will increase either following its embedment from wet-lab experimentation or as a consequence of validation of instantiated hypotheses from virtual experimentation. The embedded knowledge can be leveraged to improve and individualize the utility of biomarkers of drug-induced liver injury and to better understand the sources of interindividual variability. Model modularity across spatial and temporal scales, from metabolism and cellular damage to mouse liver and body, enables seamless translation to humans and extension of use cases to other toxicants and biomarkers.

## Methods

### Overview

Conventional pharmacokinetic and pharmacodynamic (and toxicodynamic) models focus on prediction and describe the dynamics of model components by solving systems of equations (e.g., see [6,9]). Making predictions is not an objective for this work; we have fundamentally different objectives. The following claim, supported subsequently, illustrates achieving those objectives. The actual spatiotemporal mechanisms causing APAP-induced hepatic injuries within mice following a toxic APAP dose and the virtual counterparts during execution are strongly analogous dynamically. To produce evidence that supports (or not) that claim, requires that we employ methods that are fundamentally different from those used to support conventional pharmacokinetic and pharmacodynamic modeling efforts. For example, we do not employ systems of descriptive equations. Components are concrete biomimetic objects. Time advances in discrete steps. Discrete event and agent-oriented software methods are most prominent. Accordingly, some of our terminology is also unconventional. A novel advantage of our approach and methods is the ability to represent both knowledge and ignorance concurrently, which is important for this work because multi-source uncertainties have proven to be a stubborn barrier to progress in explaining ALT release.

The evidence needed to support the above claim comes from conducting virtual experiments (vExperiments, hereafter) that challenge the ability of closely related, demonstrably biomimetic model mechanisms (MMs) to generate patterns of virtual hepatic injury that scale (or not) to be quantitatively similar to wet-lab validation targets, as detailed in subsection Experiment design. A vExperiment is a test (a trial) of an extant hypothesis, such as Hypothesis 1 in the Introduction. Executions produce simulations with features that we measure. Those measurements enable testing the hypothesis. We expand on that point in subsection Making virtual measurements analogous to wet-lab measurements. By documenting that similarities between measures of virtual features and phenomena meet the demanding set of requirements in subsection Model mechanism requirements, the results of vExperiments can be used to support the above claim. Model mechanism details during execution help bring into focus ways in which particular sources of uncertainty become entangled with cause-effect details. Observing the unfolding of events during an execution allows one to think through the MM’s networked cause-effect details and identify (or not) weaknesses. Falsification of one (or more) of the MMs features—demonstrating its inability to achieve its target phenomena—provides new knowledge and shrinks the constellation of explanatory MMs incrementally.

To distinguish virtual mouse components, characteristics, and phenomena from real mouse counterparts, we capitalize the former hereafter and, in three cases, append the prefix “v.”

### Experimental design

**Fig 1:**
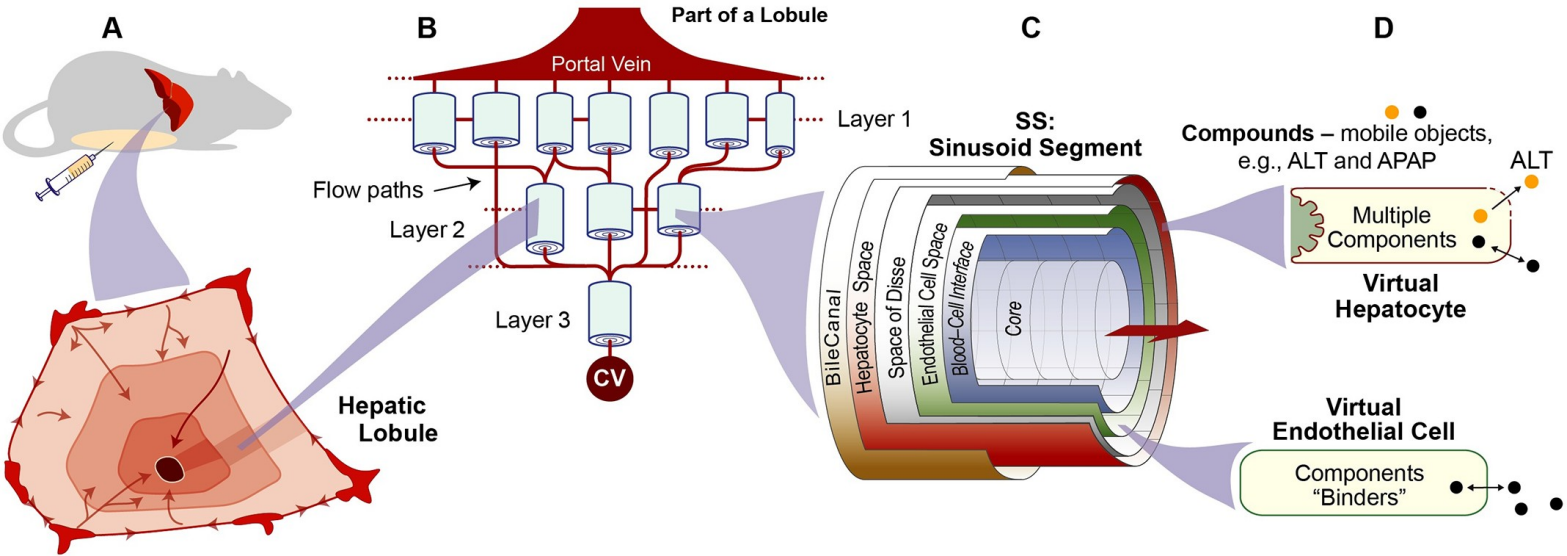
Component organization and model mechanism features. (A) A virtual Mouse, detailed in Methods, is a concretized, coarse-grain software analogy of an actual mouse. Shading within a cross-section of a hepatic lobule illustrates idealized PP-to-PC gradients. (B) A portion (16%) of one vLobule is illustrated. A Monte-Carlo specified interconnected directed graph, which can be different (within constraints) for each vExperiment, specifies flow paths for APAP and other Compounds (see Methods). (C) A multi-layered, quasi-3D Sinusoid Segment (SS) maps to a portion of hepatic tissue. One is placed at each graph node. An SS functions during execution analogous to sinusoid components and features averaged across many actual lobules; SS dimensions are Monte Carlo-sampled to mimic lobular variability. An SS comprises a Core surrounded concentrically by five 2D grids. One space contains virtual Endothelial Cells (vECs) and another contains vHPCs. (D) Mobile Compound objects move within and between the grids in C. They enter and exit an SS via Core and Interface, percolate stochastically through accessible spaces influenced by flow parameters. Compounds that exit to the CV are returned to Mouse Body. vHPCs and vECs control Compound entry from, and exit to, adjacent spaces, and the fate of Compounds within. Each vHPC contains a variety of components needed to enable the cause-effect events. All of the preceding components are parameterized the same as in Smith et al. [8].

We experiment on the Mice illustrated in Fig 1. Their components are strongly analogous to mouse counterparts within and across multiple levels, but only to the extent needed to achieve use case objectives [10]. Many of the methods used are identical to those detailed by Smith et al. [8,11]. Nevertheless, to facilitate reproducibility and support descriptions of method extensions and explanations of results, we provide abridged descriptions of methods reused herein. We utilize the vExperiment protocol outlined by Kirschner et al. [12], as recently enhanced [8,13]. Mice employ a previously validated spatiotemporal MM that explains key early features of APAP hepatotoxicity [14]. Given that the same MM is reused in testing all four ALT Externalization Hypotheses, we name it the Parent MM.

We describe vExperiment setup in S1 Text. Because of multisource uncertainties, the constellation of plausible MM configurations and parameterizations capable of achieving validation targets and Similarity Criteria is large. Thus, it is essential to conduct many narrowly focused vExperiments to incrementally shrink that constellation. To do so, we follow the Iterative Refinement Protocol (IRP) [15,16,17]. The following is an abridged description. Additional detail is provided in S1 Text.

The IRP is a scientific method for falsifying, refining, and validating explanatory MMs. The IRP begins with a MM, such as the Parent MM, which has achieved several validation targets. For an existing validation target, we specify an enhanced Similarity Criterion or a new feature or phenomenon (e.g., accumulation of ALT-in-Mouse-Body). A Similarity Criterion specifies when and how a validation target or a Target Attribute has been achieved. For example, the scaled mean measure falls within ± 1 standard deviation of the mean target wet-lab value. If needed, we make MM changes and reverify the software. We then specify the configuration and a parameterization. The test of the hypothesis is that results from using the new parameterization will achieve the new Similarity Criterion. We conduct vExperiments. Often, the results from using the initial parameterization fail to support the hypothesis. In that case, that particular MM is falsified. Such a falsification completes one IRP cycle. For the next IRP cycle, the focus turns to parameterization refinements with the expectation that, during that cycle, the Similarity Criterion will be achieved.

The iterative process of falsification-refinement-validation ensures that the MM is increasingly biomimetic yet parsimonious. The process provides explanatory insight into how, where, and when various referent features may be generated. It also means that MMs are perpetual works in progress, not finished products. MMs are improved incrementally through multiple future IRP cycles that challenge the MM and validate against an expanding set of referent measures.

### Making virtual measurements analogous to wet-lab measurements

To strengthen the virtual-to-wet-lab experiment analogy, MM features and phenomena are measured analogous to how corresponding wet-lab measurements are (or might be) made. At the conclusion of a simulation cycle, we can, for example, see the locations of all Compounds, determine whether a particular event has occurred (such a breaching a Threshold), and identify the state of each vHPC. To assign a value to some feature, we must measure it, e.g., count the number of APAP objects within all vHPCs at particular locations. Those measurements are made by agents within the framework at the end of each simulation cycle [8,13,15].

Most events are stochastic and several features are Monte Carlo-sampled at the start of an execution. Accordingly, measurements recorded during an execution can exhibit considerable variability. That variability is intentional to help account for the variability and uncertainty that characterizes wet-lab measurements. Consequently, measurements are averaged across several executions. For this work, the number of executions per vExperiment ranges from 12 (most vExperiments) to 72 (when small APAP doses are used; the smaller the dose, the greater the measurement variance between executions).

### Model Mechanism requirements

We define a mechanism, virtual or real, as entities and activities organized and orchestrated in such a way that they are responsible for the phenomenon to be explained [18]. The MMs studied herein emanate from five demanding requirements that guide software engineering, MM instantiation, and simulation refinements.

1. Five primary characteristics of a biological mechanism–During execution, a MM must exhibit the characteristics of a biological mechanism [19]. 1) For the duration of a vExperiment, the MM is responsible for a virtual phenomenon that mimics the biological phenomenon to be explained (the Target Attribute). 2) It has components (modules, entities, etc.) and activities that are 3) arranged spatially and exhibit structure, localization, orientation, connectivity, and compartmentalization compartmentation that are (based on the preponderance of the available evidence) dynamically analogous to biological counterparts. 4) Activities during execution have temporal aspects, such as rate, order, duration, and frequency. 5) The MM has a context, which can include being in a series and/or a hierarchy.
2. Biomimicry–Components and activities are biomimetic [20,21] according to pre-specified criteria, and they facilitate analogical reasoning [14,22].
3. Strong parsimony guideline–When scaled, measurements of selected features match or mimic (are strongly analogous to) prespecified Target Attributes (such as the individual plasma ALT value) to the extent needed to achieve face validation and the specific Similarity Criterion. Adhering to this guideline helps manage the number of equally plausible MMs, while enabling one to increase complexity incrementally. It also facilitates distinguishing a cause from an effect.
4. Emergence–Phenomena measured at a higher level of organization arise mostly from entanglement of local component interactions and phenomena at a lower level of organization.
5. Mobile objects–Each mobile object, such as an APAP and an ALT, maps to a small amount of their chemical counterparts. Quasi-autonomous components (i.e., software agents such as a Sinusoidal Segment (SS) and a vHPC) recognize an APAP and an ALT and adjust responses appropriately. For example, a vHPC recognizes that an adjacent APAP has the property *membraneCrossing* = true, and allows it to enter stochastically.

### Mouse components and their organization

A Mouse (Fig 1) comprises Mouse Body, vLiver, and a space to contain Dose for simulating intraperitoneal dosing. It is engineered to facilitate independent replication of experiment results. A vLiver is the number of Monte Carlo-sampled vLobule variants per vExperiment. A vLiver is engineered to be scientifically useful in a variety of usage contexts, but is not intended to (and does not) precisely model a mammalian liver. Rather, it is quantitatively and qualitatively biomimetic during execution in particular ways. It is strongly analogous to actual livers across several anatomical, lobular, and cell biological characteristics. The vLiver used for this work achieved qualitative and quantitative validation targets for several different compounds [8,23–25]. Consequently, its structure and composition are relatively stable and robust. An abridged description follows. Additional details are provided in S1 Text.

One vLobule maps to a small random sample of lobular flow paths along with the volume of associated tissue. A vLobule comprises a directed graph with a particular SS object at each graph node. Flow follows the directed graph. Graph nodes are organized into three Layers. At the start of each execution, all SS dimensions are Monte Carlo-sampled within constraints as [23,25]. Events occurring within a particular SS are dynamically analogous to referent events occurring within sinusoids and adjacent tissue. Virtual Endothelial Cell (vEC) objects occupy 99% of the vEC Space. vHPCs occupy 90% of the Hepatocyte space. An APAP object maps to a tiny fraction of an actual APAP dose. APAP Doses are ≤ 100,000 objects. Each simulation cycle (discrete time-step that scales to approximately 1 second), a fraction of APAP in Body is transferred to Portal Vein (PV). Compounds percolate stochastically through accessible spaces toward the Central Vein (CV) influenced by parameter values that control local flow. Compounds exit into CV, where they get moved to Mouse Body.

**Fig 2:**
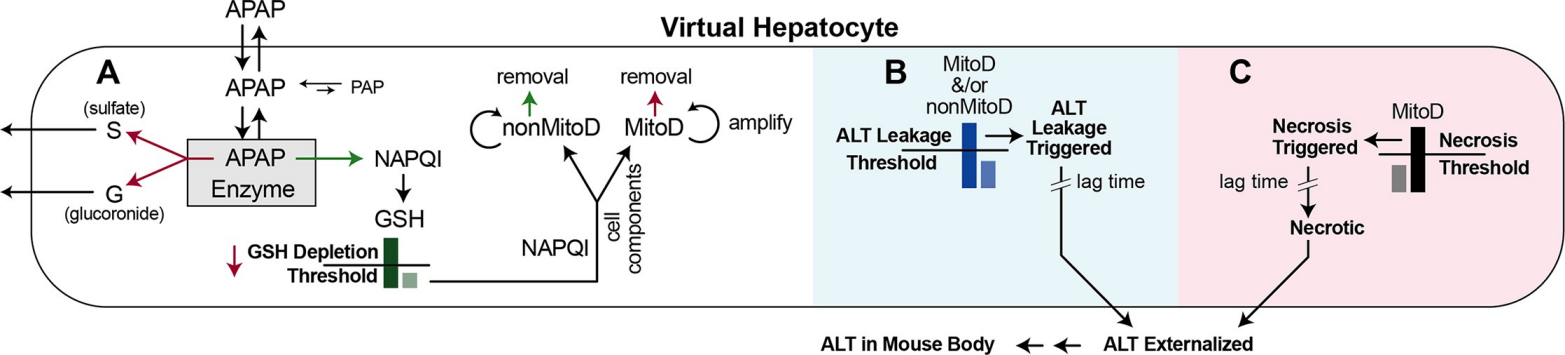
Events that can occur within each vHPC. Arrows indicate discrete probabilistic events (not continuous processes) that may occur during a particular simulation cycle. (A) These features and events are parameterized the same as in Smith et al. [8]. There is a direct mapping between the probability of an APAP Metabolism event and average metabolic capacity of hepatocytes at various PP-to-PC locations. Red arrow: event probability or value decreases PP-to-PC. Green arrow: event probability increases PP-to-PC. A NAPQI removal event depletes Glutathione (GSH). Once GSH falls below the vHPC’s GSH Depletion Threshold, each subsequent NAPQI removal event is paired with creation of one of two types of Damage Product, either a MitoD object (maps to mitochondrial damage products) or a nonMitoD object (maps to non-mitochondrial damage products). Amounts of MitoD and nonMitoD are amplified [8]. A MitoD or nonMitoD object may be removed, which maps to a damage mitigation process. The probability of a MitoD removal event decreases PP-to-PC, whereas the probability of a nonMitoD removal event increases PP-PC [8]. (B) The virtual counterparts of two types of damage-induced ALT externalization events are illustrated. 1) Accumulation of MitoD objects above an ALT Leakage Threshold triggers ALT leakage and subsequent ALT externalization. 2) Accumulation of nonMitoD objects above an ALT Leakage Threshold triggers ALT leakage and subsequent ALT externalization. Both ALT Leakage Threshold values are the same. 3) Both of the preceding processes may operate concurrently. The contributions 1–3 to explanations of the target data were investigated separately. Externalized ALT accumulates in Mouse Body. (C) Independent of the events in B, accumulation of MitoD above a Necrosis threshold triggers Necrosis, the same as in Smith et al. [8]. Once Necrosis is triggered, there is a delay—a Monte Carlo sampled lag time—before the vHPC becomes Necrotic (maps to cell death) and all remaining ALT is externalized. The minimum ALT Leakage lag time is less than the minimum lag time for Necrosis Triggered → Necrotic. Parameterizations within B and C are independent of a vHPC’s PP-to-PC location. Nevertheless, ALT Externalization within B and C is dependent on a vHPC’s PP-to-PC location because of the location-dependent events in A (red and green arrows). Externalized ALT exits a SS, enters the CV, and is transferred to Mouse Body the same as unmetabolized APAP. ALT in Mouse Body maps quantitatively to plasma ALT values.

Cells are software agents. Entry and exit of Compounds from Cells is mediated by the Cell according to the Compound’s properties. vECs contain binders that bind and release APAP (maps to non-specific binding). For a typical vExperiment, the average number of vHPCs per vLobule is 16,165. vHPCs also contain binders that are responsible for non-specific APAP binding, Metabolism (Fig 2), and the APAP ⟷ *p*-aminophenol futile cycle [26].

### Model mechanisms that may explain APAP-induced liver Injury

Events that can occur within a vHPC during each simulation cycle are illustrated in Fig 2. vHPC capabilities are identical to those used previously [8]. An abridged description follows. Additional details are provided in S1 Text. The order of events is (pseudo) randomized during each simulation cycle. The probability of an APAP Metabolism event and, subsequently, that the Metabolite is NAPQI increases PP to PC. All other Metabolites are lumped together and for simplicity are divided equally between G (maps to APAP glucuronide) and S (APAP sulfate). Each vHPC has a location-determined GSH Depletion Threshold value (Fig 2A). Decreasing the GSH Depletion Threshold counter by 1.0 maps to depleting a fraction of a hepatocyte’s available GSH. Location-dependent GSH Depletion is described in detail in [8]. A counter value = 0 maps to effective GSH depletion. Thereafter, during each cycle, there is a 50% chance that a NAPQI will be removed and replaced by (n + 1) Damage Products, one of two types: a MitoD maps to mitochondria-associated damage products; a nonMD maps to all other types of damage products, including reactive oxygen/nitrogen species [8,27]. The value of n is a pseudo-random draw from the uniform [3,6] distribution.

When the MitoD amount > Necrosis Threshold value (which = 4 for this work), Necrosis is Triggered (Fig 2C) and the vHPC is designated Necrosis-Triggered. Once the Necrosis Threshold is breached, there is a lag time (Death Delay) before that vHPC transitions to the Necrotic state, which maps to histologically distinguishable necrosis. To account for the considerable uncertainty about the timing of triggering events and histological confirmation of necrosis, the lag time is a pseudo-random draw from a uniform [Min, Max) distribution = [2, 6) h.

Hepatocytes in vivo utilize multiple lobule-location-dependent mechanisms to mitigate or reverse various types of damage. We implemented a single type of Damage Mitigation event, which results in removal of either a MitoD or nonMD. The probability of such an event is different for MitoD and nonMD and depends on the vHPC’s PP to PC location. Those probabilities were arrived at following several IRP cycles [8].

Because many MM features are inscribed with a probability distribution and executed according to Monte Carlo sampling, simultaneous, small changes (e.g., 5–10%) of several configuration values can offset each other and may produce no detectable change in measured events or outcomes. Thus, linear sensitivity studies are less informative and meaningful than complete location changes in the space of MM configurations, as performed in [8] and discussed in [21]. A complication is that significant regions of a MM’s configuration space may be non-biomimetic. For example, 1) having more SSs in Layer 3 than in Layer 2 or having the probability of NAPQI formation in Layer 1 be greater than in Layer 3 is not biomimetic. Domain knowledge is used to constrain exploration and analysis only those regions of configuration space that are known to be, or are plausibly, biomimetic.

### Limits on mappings

We seek a balance between more detailed biomimicry and the computation programmed into virtual Mice and their methods. A SS does not map directly to a portion of a single sinusoid surrounded by hepatic endothelial cells, hepatocytes, etc. Instead, as described in Hunt et al. [21], events occurring within a particular SS are intended to be strongly analogous to referent events thought to occur within portions of sinusoids and adjacent tissue. The mapping from cylindrical 2D Hepatocyte Space to corresponding 3D configurations of hepatocytes is indirect and not intended to be literal. Instead, as a MM component, it is intended to be adequately (defined in advance) analogous. Because a SS does not map directly to a portion of a single sinusoid, a vHPC cannot map 1:1 to a hepatocyte, although there is a strong functional analogy. Rather, a vHPC at a particular Hepatocyte Space grid point maps to a conflation of relevant hepatocyte functionality at a corresponding PV-to-CV location. Nevertheless, events occurring within a particular vHPC do map directly to corresponding events occurring within hepatocytes at a comparable location.

### Technical details

Mice were acquired, cared for, and treated as described in the original studies [7].

The Java-based MASON multiagent toolkit serves as the basis for virtual Mice and many of their components. In earlier work, we referred to virtual Mice as Mouse Analogs (Liver Analogs, Hepatocyte Analogs, etc.) to stress the fact that MM entities are intended to be strongly analogous to their biological counterparts. They do not precisely model the biology. Hunt et al. characterize the spectrum of mechanism-oriented model types being used to help explain biological phenomena [28]. We utilize agent-oriented modeling methods and techniques [29], which allow for complex software entities to be biomimetic in multiple ways. All vExperiments were run using local hardware and virtual machines [30] on Google Compute Engine, running 64-bit Debian 9. Quality assurance and control details, along with practices followed for validation, verification, sensitivity analyses, and uncertainty quantification are as discussed in [8].

To support verification and validation efforts, all vExperiments include a Marker Compound as part of Dose. It acts as a virtual internal standard. It behaves analogous to sucrose in vivo and serves as an indicator of vLobule-structure-Disposition interaction. Absent structural and vLiver component changes, Marker behavior during executions is invariant. Use of Marker is explained further in S1 Text.

A Mouse is treated as a form of data, using both the implicit schema of Java, JavaScript, and R and the explicit schema of its configurations. Mice and configuration files are managed using the Subversion version control tool in two repositories, one private (Assembla) and another public. The R programming language is used to facilitate analysis and plotting vExperiment measurements. Values for key vHPC specifications and parameterizations are listed in S1 Table. The entire toolchain, including the operating system, configurations, and I/O handling is open-source. The data presented herein, along with the code, are available from project websites (https://simtk.org/projects/aili andhttps://simtk.org/home/isl/), and are available to be licensed as open data.

## Results

For the results that follow, parameterizations of components in Fig 1, and events in Fig 2 are unchanged from Smith et al. [8]. Parameterizations for key features are listed in S1 Table. Achieving the validation targets for this work without having to alter the Parent MM strengthens the case that the Parent MM is strongly analogous to the actual APAP-induced hepatotoxicity mechanism.

### Two ALT release processes: one confirmed, the other plausible

Evidence suggests that externalization of damaged macromolecules is a normal, ongoing hepatocyte process. Additional externalization processes may (or not) be engaged as rates of damage product accumulation increase and the nature of those products change. Consequently, undamaged macromolecules such as ALT may become coupled to one or more of these processes and become externalized directly or concomitantly.

**Fig 3:**
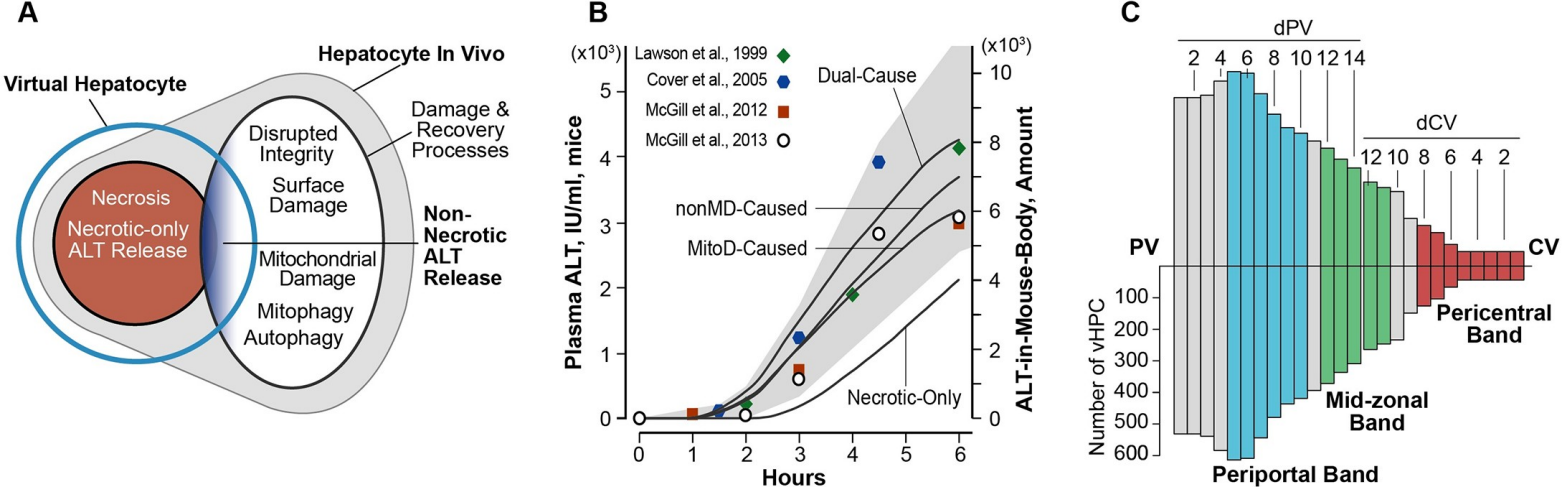
ALT externalization processes, validation targets, and Lobular organization. (A) An illustration of plausible relationships between the ALT externalization processes in Table 1 and in vivo counterparts. Non-Necrotic ALT Release = (MitoD-Caused + nonMD-Caused + Dual-Cause) and maps to a structured conflation of non-necrotic damage and recovery processes that may directly or concomitantly enable ALT release, indicated by blue shading. (B) The gray area is the initial validation target range (left axis), which is based on mouse data from the four indicated reports. The minimal Similarity Criterion for an acceptable ALT release MM is that it generates ALT-in-Mouse-Body profiles that, when scaled (right axis), fall within the target range. (C) Bar heights represent the mean number of vHPCs at the indicated location within an average vLobule. The left edge corresponds to PV. The right edge corresponds CV. Moving left-to-right, the first 14 bars are located at increasing distances from PV (designated dPV) along the average PV-to-CV path. Moving right-to-left, the first 12 bars are located at increasing distances from the CV (designated dCV) along the average CV-to-PV path. Periportal band = blue bars, Mid-Zonal (M-Z) band = green bars, and Pericentral band = red bars.

The parsimonious working hypothesis, illustrated in Fig 3A, is that ALT may be released by two separate processes, passive release as a consequence of cell death and non-necrotic release processes. The supporting evidence is circumstantial. Gamal et al. described leakage of cytoplasmic material associated with APAP-induced disruption of hepatocyte integrity and surface damage associated with blebbing [31]. Ni et al. reported that hepatocytes upstream of necrosis experience hepatocellular damage mitigation, including evidence of autophagy and mitophagy [32]. Following a subtoxic APAP dose, APAP-protein adducts are externalized to blood in the absence of necrosis [7,33]. If a normal cellular process is damaged during APAP-induced injury, its selectivity may be eroded leading to concomitant externalization of other macromolecules, including ALT. Consistent with that scenario, McGill et al. reported that, following a low toxic APAP dose, APAP-protein adducts are externalized to blood prior to ALT elevations [7].

We conjectured that instantiating a virtual counterpart to non-necrotic ALT release processes should be feasible without requiring any change to the Parent MM because the two types of damage products generated (MitoD and nonMD) map to a coarse grain conflation of all types of hepatocellular damage that could lead to ALT release.

### Hypothesis 1, Necrotic-only ALT externalization

The Parent MM includes transitioning a vHPC from the Necrosis-Triggered state to the Necrotic state following a Death Delay lag time. Adding ALT release as a consequence of that transition was straightforward. We added ALT objects to each vHPC along with the instruction that any remaining ALT objects are released when the vHPC becomes Necrotic. Because there is no strong wet-lab evidence to the contrary, we assumed that each hepatocyte, independent of lobular location, contains the same amount of ALT. Using ALT per vHPC = 5 objects at t = 0 proved sufficient. One ALT object maps to a small amount of actual ALT. When a vHPC transitions from Necrosis-Triggered to Necrotic, all remaining ALT is released and externalized (bottom, Fig 2). Externalized ALT objects follow the same stochastic movement rules as APAP, G, and S. Upon entering the CV, an ALT is moved to Mouse Body. An ALT in Mouse-Body is scaled directly to represent plasma ALT. We name that entire sequence, from Metabolism to ALT externalization, Necrosis-only. It corresponds to Hypothesis 1 in the Introduction. Following a tissue distribution phase, the plasma half-life for ALT in mice is approximately 25 h [34]. During the first 6 h of experiments in mice, we conjecture that little of the externalized ALT is cleared. For simplicity, ALT in Mouse-Body is not removed. It accumulates and does not re-enter the vLiver.

### Alternative Hypotheses 2–4, explaining ALT externalization using three MM variants

The early results in Fig 3B showed that ALT externalization and accumulation in Mouse Body as a consequence of the Necrosis-only MM could not scale quantitatively to closely match the initial validation targets pooled from four reports. Accordingly, we sought parsimonious extensions of the Parent MM that 1) would map to the non-necrotic ALT release process, 2) would operate concurrently with—but independent of—Necrosis-only, and 3) make ALT Externalization a direct function of Damage Products. We instantiated three versions of the coarse grain non-Necrotic ALT Release (Fig 3B) by making ALT externalization a direct function of amount of Damage Products in each vHPC: nonMD, MitoD, or both. They are listed in Table 1.

**Table 1:** Descriptions of four processes that may cause ALT externalization.

Name	Within the model, ALT is released:	Corresponding in vivo hypothesis: ALT release is a consequence of:
Necrotic-only	upon transition from Necrosis-Triggered to Necrotic	hepatocyte death (Hypothesis 1)
MitoD-Caused	after the ALT Leakage Threshold for MitoD is exceeded^1^	mitochondrial damage with or without hepatocyte death (Hypothesis 2)
nonMD-Caused	after the ALT Leakage Threshold for nonMitoD is exceeded^1^	general cell damage with or without hepatocyte death (Hypothesis 3)
Dual-Cause	after both of the above Leakage Thresholds are exceeded^1^	concurrent mitochondrial and general cell damage with or without hepatocyte death (Hypothesis 4)

^1^ For some (especially PC) vHPCs, the Necrosis Threshold will also be triggered. For those vHPCs, all remaining ALT is released later, when the vHPC transitions to Necrotic.

Four parameters govern ALT externalization from the MitoD-Caused MM, the nonMD-Caused MM, and the Dual-Cause MM. 1) The initial amount of ALT per Cell, 2) the Damage or Leakage Threshold, 3) the minimum lag time, and 4) the maximum lag time. Parameters 3 and 4 were the focus of several IRP cycles. The initial amount of ALT per vHPC is a constant, and controls the plateau value of ALT in Mouse Body. ALT per vHPC and Leakage Threshold are not varied to achieve validation. The relationship between parameters 1 and 2 is noteworthy, because it is mediated by the amount of MitoD and/or nonMD. If the Leakage Threshold had been larger, e.g., 5 rather than 1, we could still arrive at the same ALT externalization phenomena, but it would have required increasing the amplification of nonMD and MitoD. However, doing so would have provided no new insights while adding computational costs. If the initial amount of ALT per cell had been larger, we could also achieve the same validation targets, but we would need to increase Leakage Threshold and increase nonMD/MitoD amplifications.

During each simulation cycle during execution of the MitoD-Caused, nonMD-caused, or the Dual-Cause MM, every vHPC determines if its amount of nonMD and/or MitoD exceeds the ALT Leakage Threshold value. If so, that vHPC becomes Leakage-Triggered and initiates the ALT externalization process. In the MitoD-Caused (nonMD-Caused) MM, only the amount of MitoD (nonMD) within each vHPC is compared to the Leakage Threshold value. In the Dual-Cause MM, the sum of nonMD and MitoD is compared to the Leakage Threshold. Results from vExperiments to achieve stringent the validation targets (described below) used MitoD-Caused MM because we judged it to be the more parsimonious of the three. We also observed no explanatory advantage when using nonMD-Caused or Dual-Cause MMs.

The vHPC specifies a Leakage lag time by a pseudo-random draw from a uniform [Min, Max) distribution. When the lag time duration is reached and the vHPC’s ALT counter value is > 0, the vHPC creates an ALT object, externalizes it, and decrements its ALT counter by 1. An externalized ALT in “Blood” exits the CV by following the same movement rules used by APAP Metabolites.

We could have added five ALT objects to each vHPC at t = 0. Although not biomimetic, creating ALT objects as they are needed is computationally more efficient. Tracking objects in all vHPCs that are doing nothing is computationally inefficient and increases the duration of executions. Once an ALT externalization is scheduled, it will occur during that simulation cycle if the ALT counter value is > 0. After an ALT externalization is scheduled, the designated Damage Products may fall below the Leakage Threshold. When that occurs, the vHPC is no longer Leakage-Triggered.

We conducted exploratory vExperiments using Leakage Threshold values ≤ 5. Setting the Threshold = 1, independent of PP-to-PC location, enabled achieving validation targets for the Necrosis-only MM, when a Necrosis-Triggered vHPC transitions to Necrotic, and its ALT counter value > 0, the vHPC creates ALT objects corresponding to the counter value and externalizes them with a non-biomimetic zero-time delay.

### Specifying the initial validation target

Although the four MM variants in Table 1 are coarse grain analogies of the referent processes, the constellations of features and parameterizations that might merit exploration is large, and there are too few wet-lab data to guide selecting particular parameter value combinations. Thus, an essential early task was to identify a subset on which to focus. To do so, we specified a broad, semiquantitative validation target: the temporal trend of ALT-in-Mouse-Body amounts must exhibit a sigmoidal shape and fall within the target area in Fig 3B. Specification of the target region’s upper and lower bounds was guided by a selection of mean plasma ALT values from four mouse studies of toxic APAP doses [7,35–37]. Two studies used C3Heb/FeJ mice [35,36] and two used C57BL/6 mice [7,37]. Three studies used a 300 mg/kg APAP dose, whereas one used a 500 mg/kg dose [35]. Results from the 500 mg/kg APAP studies were included because there is considerable variance among mouse strains in sensitivity to APAP-induced hepatotoxicity, and we hypothesize that, when responding to a comparably toxic APAP dose, a common mix of mechanism features governs ALT release and its accumulation in plasma.

To acknowledge the variability within and between studies, we specified that the upper and lower edges of the target region shall include the mean plus or minus the value of 1 standard-error-of-the-mean, respectively, for the 3-to-6 plasma ALT values.

### Intralobular location-dependent measurements

Because the temporal pattern of upstream events within a vLobule can significantly influence the temporal pattern of downstream events, we measure features within the three bands illustrated in Fig 3C.

During each execution, there is a large variety of PV-to-CV paths that an APAP object might travel. The distribution of mean PV-to-CV distances is significantly skewed toward longer distances to validate against single-pass liver perfusion data for multiple drugs (e.g., see [23]). We use the length of the average upstream Layer (in Core grid points) to specify the distance of each vHPC from PV (designated dPV). Averaging over the Monte Carlo executions within a vExperiment, we determine the mean number of vHPCs at each dPV location. However, because we are interested in the temporal order of PC events, we also identify vHPC locations in terms of distance from CV. The distribution of CV-to-PV distances is different from the PV-to-CV distribution because the SSs in each layer can have different lengths and one upstream SS can link to multiple downstream SSs. Nevertheless, we specify the location of each vHPC along the average SS path as a distance from CV (designated dCV). At each dCV location we determine the mean number of vHPCs. Fig 3C combines a visualization of the mean number of vHPCs at dPV locations 1 through 14, which accounts for 85.3% of the average number of vHPCs per vExperiment, with mean number of vHPCs at dCV locations 1–12, which accounts for 14.1% of the average number of vHPCs. The combined visualization accounts for 99.4% of average total vHPCs. On average, for the MitoD-Caused MM, the PP band (blue bars) includes 4772 vHPCs, the M-Z band (green bars) includes 1721 vHPCs, and the PC band (red bars) includes 906 vHPCs.

During each execution, we measure amounts of APAP, G, S, NAPQI, nonMD, and MitoD in each vHPC along with counts of the following events: GSH Depletion, Damage Mitigation, Leakage-Triggered (defined below), ALT externalized (described below), Necrosis-Triggered, and Necrotic. Those values are summed or averaged over all Monte Carlo executions for the whole vLiver and within the three bands.

### APAP disposition and toxicity measurements

APAP disposition and toxicity measurements for all four MMs in Table 1 are identical within the variability of Monte Carlo executions. They are also essentially the same as those reported by Smith et al. [8]. For convenience, the following profiles are provided in S1 Fig: APAP and its Metabolites, G and S, in Mouse Body; average amount of APAP per vHPC within the PP, M-Z, and PC bands identified in Fig 3C; average amount of NAPQI within PP, M-Z, and PC bands; distance from CV of average Necrosis-Triggered events within PC and M-Z bands; the cumulative GSH depletion events; and cumulative Damage Mitigation events.

**Fig 4:**
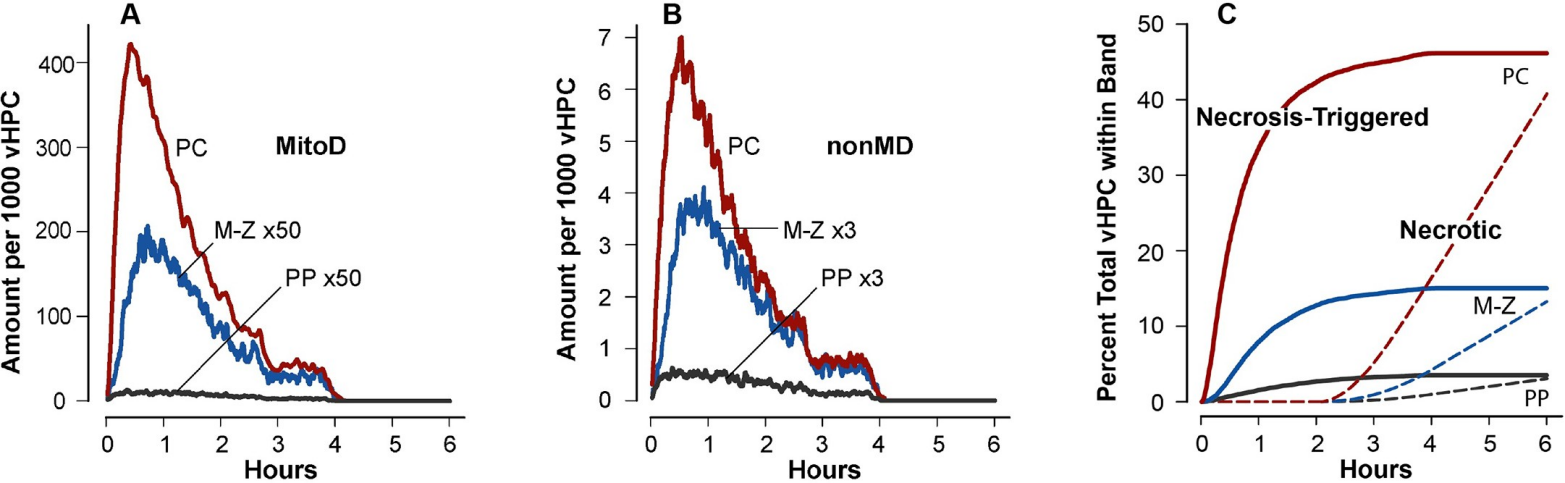
Temporal measurements made during executions of the Parent Model Mechanism. Average amount of MitoD (A) and nonMD (B) within PP, M-Z, and PC bands. (C) Cumulative Necrosis-Triggered and Necrotic events (dashed curves) within the three bands. vHPCs that are Necrosis-Triggered become Necrotic following a Monte Carlo sampled Necrosis interval.

The amount of MitoD per vHPC within M-Z and PP bands (Fig 4A) is minute compared to the amounts within the PC band. Three MM features (see S1 Fig) help explain the differences. 1) The probability that an APAP Metabolism event will generate a NAPQI is smaller in M-Z and PP bands than in the PC band. 2) The GSH depletion threshold, which maps to relative amounts of GSH, decreases PP-to-PC. 3) A more influential feature is the probability of a MitoD or nonMD Damage Mitigation event. For MitoD, it is larger in PP and M-Z bands than in the PC band, whereas for nonMD, it is smaller in PP and M-Z bands than in the PC band. Consequently, the amounts of nonMD within a vHPC in the PC band (Fig 4B) are much smaller than MitoD amounts. Within the PC band, 50% of Necrosis-Triggered events occur within the first 40 minutes (Fig 4C). Recall that the minimum delay (Fig 4C) before a Necrosis-Triggered vHPC becomes identifiable as Necrotic is 2 h.

### Quantitative mapping of ALT-in-Mouse-Body to individual plasma ALT values

The most stringent validation targets are the plasma ALT values from 18 matched mice that received the same toxic APAP dose during the same experiment [7]. To support a strong explanatory analogy, a MM must use the following direct mapping functions. At each measurement time, *t*, ALT objects in Mouse Body will scale directly to a fixed concentration (amount) of ALT in small aliquot of plasma (and vice versa). Specifically,

$$Y_{t}=S\cdot X_{t}+\epsilon$$ (1)

where *Y*_*t*_ = the mean concentration of ALT in plasma (IU/ml) at time *t*; *X*_*t*_ = the mean number of ALT objects in Mouse Body at *t* simulation cycles after Dosing; *S* is the scaling constant, and *ϵ* is random error. To account for individual variability, we hypothesize that a plasma ALT measurement at time *t* for an individual mouse is also directly proportional to the mean total ALT-in-Mouse-Body, as specified by

$$y_{i,t}=\delta_{i}\cdot (S\cdot X_{t}+\epsilon )$$ (2)

where *y*_*i*,*t*_ = the measured concentration of ALT in plasma (IU/ml) for mouse *i* at time *t* after dosing, and *δ*_*i*_ = the degree to which a mean ALT-in-Mouse Body amount must be skewed (amplified or diminished) to match the individual’s plasma ALT value. *δ*_*i*_ is applied to (*S*⋅*X*_*t*_ + *ϵ*) because it seems infeasible to separate the individual random error.

The Individualized Mapping Criterion is most stringent. It specifies that *δ*_*i*_ should be distributed somewhat symmetrically with mean = 1.0 (± tolerance) for all 18 mice. Absent comparable work to provide guidance, we started by setting tolerance arbitrarily at ± 0.1.

*S* and *δ*_*i*_ map the profile shapes between ALT-in-Mouse-Body amounts and plasma ALT values, establishing the behavioral analogy. Having drawn both structural and behavioral analogies, the limitations discussed below are mitigated enough to suggest further mouse studies.

### Kinetics of ALT externalization

Elevated plasma (or serum) ALT values are seen in humans and occasionally in mice following APAP doses considered to be nontoxic. From that, we inferred that the lag time between formation of NAPQI reaction products (or NAPQI-induced damage products) and ALT externalization must be shorter than the lag time between triggering necrosis and confirming necrosis histologically. However, we lacked information to guide specification of the Min-Max range of the Leakage lag time distribution.

Results of early explorations showed that ALT-in-Mouse-Body amounts are particularly sensitive to values chosen for Leakage lag time. However, we had no information to guide specification of the lag time distribution. Selection of a plausible minimum value was guided by whether scaled ALT-in-Mouse-Body profiles fell within the initial target range in Fig 3B. We explored a variety of distribution ranges. By using the uniform distribution with [Min, Max) = [2700, 18000) simulation cycles, which scales to [0.75, 5] h (mean = 2.88 h), and a narrow range of Eq 1 *S* values, we were able to meet the initial target range criterion for the MitoD-Caused, the nonMD-Caused, and the Dual-Cause MM, but not the Necrotic-only MM. We continued using that range for the duration of this work.

**Fig 5:**
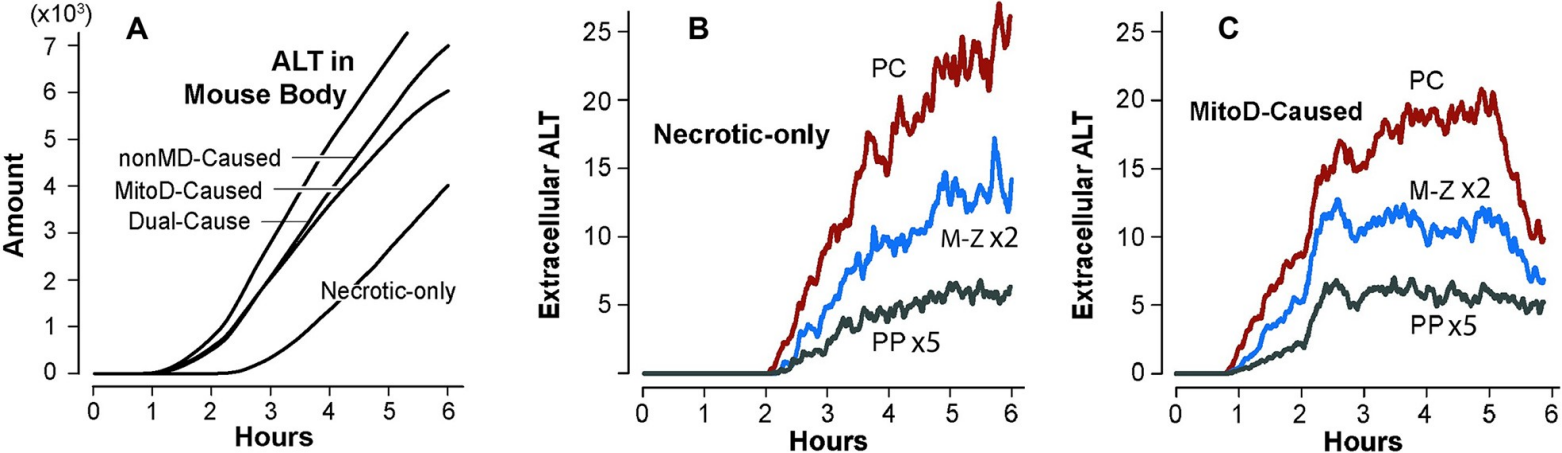
ALT Release characteristics and features during MM executions. (A) Average ALT accumulation in Mouse Body from vExperiments using each of the four MMs in Table 1. (B) The values are the average amounts of ALT that have been externalized by vHPCs within the three bands (Fig 3C) during the vExperiment using the Necrotic-only MM. However, at the time measured, that ALT has not yet exited the band. (C) Average ALT amounts, as in B, from vExperiments using the MitoD-Caused MM. The relative patterns of corresponding measures from vExperiments using the nonMD-Caused and the Dual-Cause MMs are similar to those in C.

Although the disposition of APAP and the occurrence of Damage is identical among the four MMs, ALT release is different (Fig 5A). Accumulation of ALT in Mouse Body from the Necrotic-only MM differs from that of the three other MMs because the lag time from a Necrosis-Triggered event to transition to Necrotic is longer than the lag time from an ALT-Leakage-Triggered event to an ALT Externalization event.

ALT enters Mouse Body several time steps after it is externalized and becomes Extracellular within the vLobule. Measures of Extracellular ALT are provided in Fig 5B and 5C. Some ALT is scheduled to be released within a few minutes after Dosing. Measures of ALT that is scheduled to be released are provided in S2 Fig. By 30 min post-Dose, about 50% of vHPCs within the PC band have experienced a Leakage-Triggered event.

Any meaningful change within the networked processes from Metabolism to Damage production and Damage Mitigation to ALT externalization can impact the shape of the resulting Body profile. Changes in Histological details, such as SS features (e.g. Cell spaces/densities, Space of Disse) along with dimensions and flow path connections, can also alter the ALT-in-Mouse-Body profile, absent any changes in events occurring within vHPCs.

### Individualized model mechanism-based explanation for plasma ALT values

From a hepatotoxicity perspective, we reasoned that the MitoD-Caused MM is marginally more parsimonious than the nonMD-Caused MM because MitoD production, rather than nonMD production, is directly correlated with observed PC patterns of tissue damage [7,8]. Further, the MitoD-Caused MM is more parsimonious than The Dual-Cause MM because ALT Leakage-Triggered within the latter is dependent on both categories of damage product. Accordingly, we used the MitoD-Caused MM to seek Eq 2 parameterizations capable of achieving the Similarity Criterion for mapping ALT in Mouse Body to individualized plasma ALT values. Should it be needed, the same method can be used to seek Eq 2 parameterizations for the nonMD-Caused MM and the Dual-Cause MM that also achieve the Similarity Criterion.

**Fig 6:**
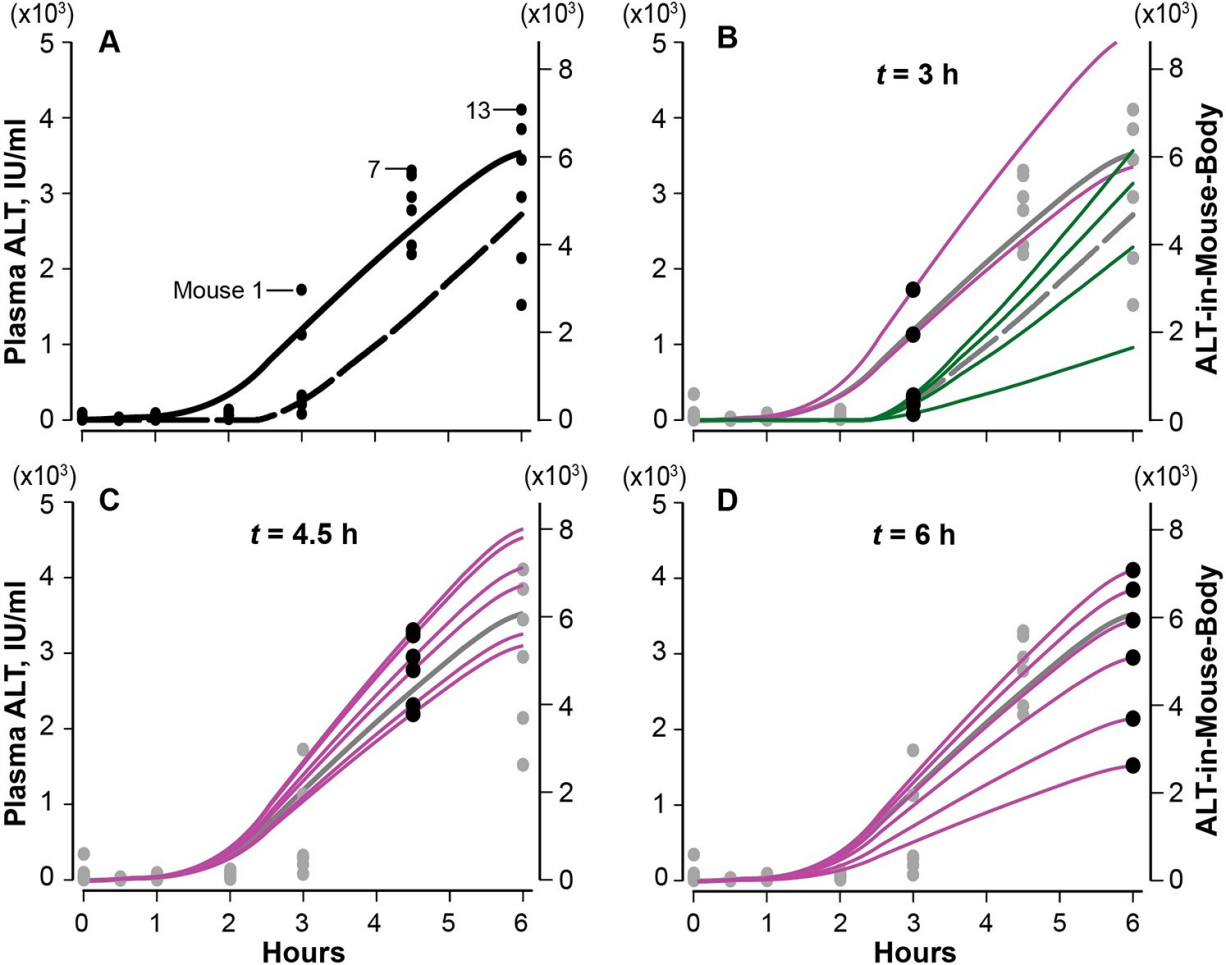
Results from the MitoD-Caused MM vExperiments are scaled to match plasma ALT values from 18 individual mice. (A) Plasma ALT values from groups of six mice are plotted at the times indicated. We targeted the 18 mice at 3, 4.5, and 6 h; they are numbered in sequence (from largest to smallest at each time). Solid curve: average amounts of ALT-in-Mouse-Body (right axis) from the MitoD-Caused MM vExperiment. Dashed curve: average amounts of ALT-in-Mouse-Body from the MitoD-Caused_exLT_ MM vExperiment (exLT = extended lag time); in that vExperiment, the [Min, Max) distribution for the ALT Release and the Death Delay lag times are increased by 1600 s (26.67 min). Both curves: ALT amounts are scaled to plasma ALT values (left axis) using Eq 1, with S = 1.72 (IU ml^-1^, ALT-objects^-1^). (B) The validation targets are the six 3 h values (black); the other values are gray. The solid and dashed gray profiles are the same as in A. Purple profiles: the average ALT amounts from the MitoD-Caused MM vExperiment in A are scaled to match the individual plasma ALT values from mice 1 and 2 using Eq 2. Green profiles: the average ALT amounts from the MitoD- Caused_exLT_ MM vExperiment in A are scaled to match the individual plasma ALT values from mice 3–6 using Eq 2. (C) and (D) The average amount of ALT-in-Mouse-Body from the MitoD-Caused MM vExperiment in A are scaled using Eq 2 to match the individual plasma ALT values from mice 7–12 (C) and 13–18 (D). The *δ*_*i*_ values for all 18 mice are listed in S2 Table. Statistical measures for the 12 Monte Carlo MitoD-Caused MM trials prior to scaling using Eq 1 are provided in S3 Table.

At the start of the scaling workflow, we encountered an obstacle. The variability in plasma ALT values at 4.5 and 6 h in Fig 6 are consistent with commonly encountered interindividual variability in APAP-induced hepatotoxicity. However, the clustering of plasma ALT values at 3 h (one subgroup of four mice; a second subgroup of two mice) suggests an additional influence that is not evident (or absent) in the mice measured at 4.5 and 6 h. We temporarily set aside the values for mice 3–6, and focused on the plasma ALT values from the remaining 14 mice. We determined that using *S* = 1.72 achieved the Individualized Mapping Criterion: we obtained sample mean, $\bar{x}$ = 1.016, standard deviation, *s* = 0.277, and coefficient of variation = 0.274. The individualized mappings are shown in Fig 6B–6D and the *δ*_*i*_ value for each mouse are listed in S2 Table.

We tested the hypothesis that shifting the [Min, Max) of the Leakage lag time distribution to larger values, without changing other parameterizations, would enable the four smallest 3 h plasma ALT values to also achieve the Similarity Criterion. Results support that hypothesis. We do not know whether the trigger causing the clustering among the 3 h mice also influenced the timing of necrosis-related histopathology. For consistency, because they may have common causes, we specified that the [Min, Max) distribution ranges for both Leakage Delay and Death Delay be extended the same. The resulting MitoD-Caused MM variant is designated MitoD-Caused_exLT_. We explored several distribution extensions using *S* = 1.72. We achieved the Eq 2 individualized Similarity Criterion using a Leakage lag time distribution [Min, Max) = [4300, 19600) ([1.19, 5.44) h; median = 2.875 h) and Necrosis distribution [Min, Max) = [8800, 23200) ([2.44, 6.44) h). Combining the four *δ*_*i*_ for MitoD-Model_exLT_ (listed in S2 Table) with the 14 *δ*_*i*_ for MitoD-Model, we obtained $\bar{x}$ = 0.992, *s*^*2*^ = 0.0922, *s* = 0.3036, and a coefficient of variation = 0.3037.

### Dose-response comparisons: ALT-in-Mouse-Body and plasma ALT values

McGill et al. also reported early plasma ALT values following APAP doses of 15, 75, 150 and 600 mg/kg [7]. The MitoD-Caused MM, parameterized as in Fig 6, failed to produce reasonably similar ALT-in-Mouse-Body amounts when dosed with comparable larger and smaller Doses.

**Fig 7:**
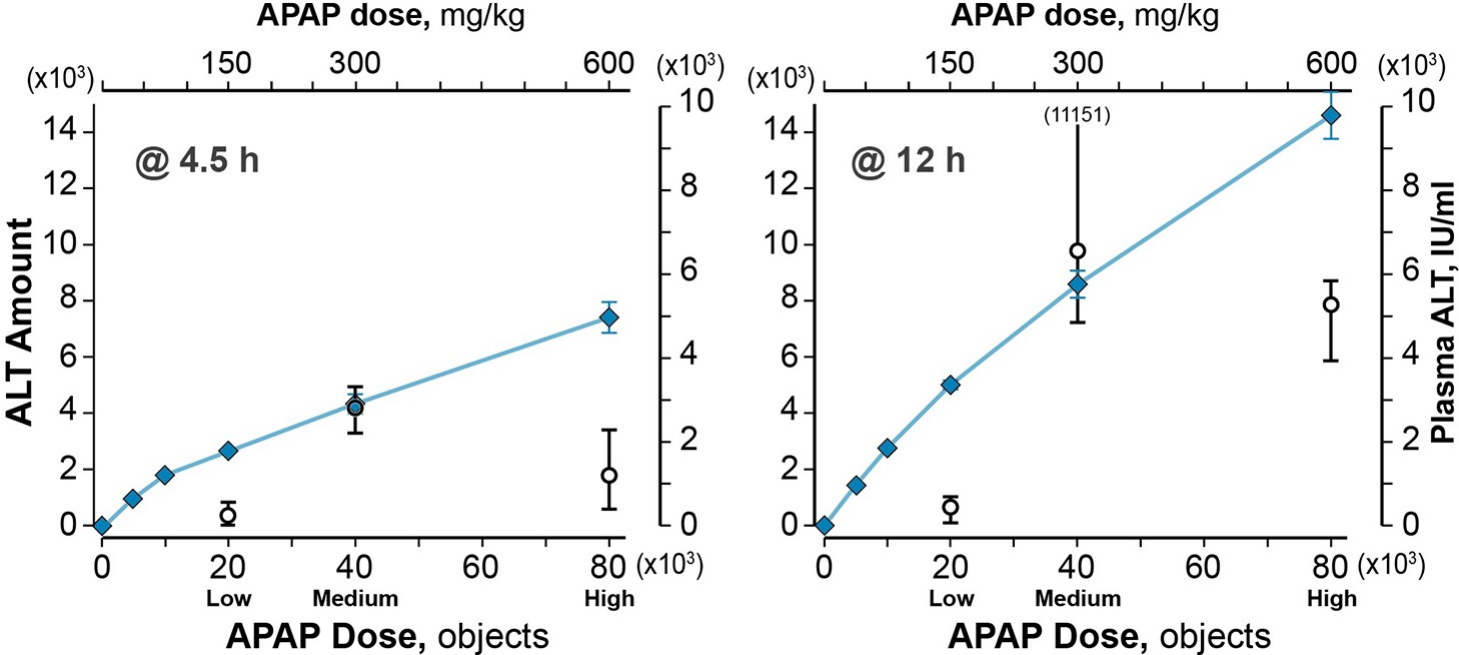
Dose-response relationships from vExperiments using the MitoD-Caused MM. Mean ALT-in-Mouse-Body amounts were recorded at 4.5 and 12 h post-Dose. The low, medium, and high Doses map directly to wet-lab doses (upper axis) of 150, 300, and 600 mg/kg, respectively. Blue diamonds: mean amounts of ALT. Whiskers for the medium, and high Doses span the range of ALT amounts for 12 Monte Carlo trials. For smaller Doses, the symbol eclipses the whiskers. Open circles: mean plasma ALT values (right axis) for three APAP doses from McGill et al. [7]; the whiskers span the range of plasma ALT values for all six mice. We map the ALT-in-Mouse-Body to plasma ALT values using Eq 1 with S = 1.72 (IU ml^-1^, ALT-objects^-1^). At 12 h, one of the six plasma ALT values is off-scale.

Measures for Necrosis-Triggered and Necrotic events from Dose-response vExperiments using the MitoD-Caused MM are provided in Fig 7. For the “low” and “high” Doses, which correspond to the 150 and 600 mg/kg APAP doses, respectively, the relative temporal patterns within the three Lobular bands are essentially the same as those in Figs 4 and 5 (and S1 and S2 Figs). Focusing on the result at 4.5 and 12 h post-Dose, scaled ALT-in-Mouse-Body amounts considerably overestimated the corresponding mean plasma ALT values for the “low” and “high” Doses. Corresponding cumulative counts of Necrosis-Triggered and Necrotic events are provided in S3 Fig Clearly, the MitoD-Caused MM provides an inadequate explanation for results of both low and high dose experiments. However, while keeping the parameterization of Parent MM features fixed, we have a variety of options to improve similarities. We knew that the temporal patterns of ALT-in-Mouse-Body amounts are sensitive to changes in the ALT Leakage Threshold, the Leakage lag time, and Death Delay, so that is where we focused.

To overcome the low-Dose mismatch, we considered several speculative scenarios. For example, within each vHPC, we could make the value of the ALT Leakage Threshold depend inversely on peak amounts of Damage Products (Fig 4A and 4B). There is a huge variety of such seemingly plausible scenarios, yet there is insufficient evidence to shrink the set. To gage how effective such a change might be, we determined the consequences of simply executing the MitoD-Caused MM variants in which ALT Leakage Threshold values were increased and decreased. Increasing the Threshold value from 5 to 10 reduced non-Necrotic ALT Release by 88% (90%) at 4.5 h (12 h) and lowered ALT-in-Mouse-Body amounts. However, the reductions were inadequate. Scaled ALT-in-Mouse-Body amounts still overestimated the corresponding mean plasma ALT values because the duration of the Necrosis was unchanged. Necrotic ALT Release—although small—still occurred and that source of ALT significantly buffered the reduction in total ALT that could be achieved by increasing the Leakage Threshold from 5 to 10. Various combinations of increased ALT Leakage Threshold, increased Leakage lag times, and increased Death Delays can be equally effective in improving similarities. However, absent evidence-based constraints and new validation targets, having a large variety of equally plausible MM variants provides no further improvement of explanatory insight.

We can improve similarities at the high Dose using a combination of increased Leakage lag times and increased Death Delays, but here too, having a large variety of equally plausible MM variants provides no further improvement of explanatory insight. Important new knowledge will emerge from discovering plausible MMs capable of explaining plasma ALT values equally well following all three doses (along with already achieved validation targets). Such MMs will be significantly more complicated than the MitoD-Caused MM. The constellation of those MMs is enlarged dramatically, relative the MitoD-Caused MM, and cannot be reduced systematically absent additional wet-lab evidence to serve as new validation targets [15,38].

## Discussion

The four MMs in Table 1 are necessarily complicated. Model mechanism details during execution are entangled within and among levels of virtual Mouse and vLiver organization (Fig 1). To support clarity in this discussion, we cluster the properties, features, and characteristics of mice (real and virtual) during experiments (real and virtual) into four groups: components, their organization and arrangements (**I**); events, their prerequisites, influence, frequency, order, and sequence (**II**); measurements and measures (**III**); attributes and generated phenomena (**IV**). For all four MMs, we have complete, detailed knowledge of **I**. The measures of **IV** can be as rich as needed. Execution produces an observable MM that meets the rigorous definition provided in subsection Model mechanism requirements. When measures of **IV** fail to meet prespecified qualitative and quantitative Similarity Criteria, we can discover and explain where, when, and why the failure occurred. Upon achieving the Similarity Criteria, the simulation stands as a challengeable yet tested MM-based theory about abstract, plausible mechanisms (**I**–**IV**) that may have occurred within individuals during the wet-lab experiments. By incrementally strengthening the analogies within **I**, **III**, and **IV**, we provide new knowledge and strengthen the analogies within **II**.

We draw several inferences from the results. Increased plasma ALT values, following a toxic APAP dose in the targeted mouse experiments are explained best by the MitoD-Caused MM, in which ALT Release is driven by both Non-Necrotic Damage and Necrosis, but not by Necrosis alone. The relative temporal contribution of Necrosis and Non-Necrotic Damage during execution to ALT-in-Mouse-Body amounts (**IV**) changes as simulation time progresses. From a biomarker perspective, it is instructive to note that their relative temporal contributions cannot be inferred from amounts of ALT in Mouse Body alone.

To match four of the 18 individually targeted plasma ALT values in Fig 6, we extended their ALT Leakage lag times and Death Delay intervals (Fig 6B), while keeping their APAP-induced Hepatotoxicity details unchanged. We infer that the in vivo counterparts of Leakage lag time and Death Delay are sensitive to unidentified non-genetic influences, such as environmental stressors (e.g., within-cage conflict). Such influences limit the reliability of ALT as a biomarker of drug-induced liver injury and likely contribute to interindividual variability. Such influences may also help explain elevated plasma ALT values absent toxicity.

We limited explorations to extensions of the Parent MM that were suggested by hypothetical mechanistic scenarios described in the literature. By doing so, we constricted to a manageable size the constellation of plausible MMs that merited exploration while adhering to the strong parsimony guideline. It is noteworthy that we achieved the stringent validation targets in Fig 6 without requiring any changes to the Parent MM. Doing so corroborates our claim that the actual spatiotemporal mechanisms causing APAP-induced hepatic injuries within mice and the virtual counterparts during execution of the Parent MM are strongly analogous dynamically. Given the results presented, we extend that claim: at comparable levels of mechanism granularity, the actual spatiotemporal mechanisms causing APAP-induced hepatic injuries in the test mice and giving rise to the targeted plasma ALT values were strongly analogous to model mechanism counterparts within the MitoD-Caused MM during execution.

The explanatory power of the MitoD-Caused MM following the medium APAP Dose (scales to 300 mg/kg in mice) is significantly eroded for Doses corresponding to 150 and 600 mg/kg of APAP (Fig 7), indicating that the unfolding and entanglement of crucial temporal features of the mechanism (**II**) in mice is predicated on APAP dose.

We detailed general weaknesses, limitations, strengths, and benefits of both the approach and methods in previous reports [10,12,28]. Reliance on analogical arguments and reasoning is both a limitation and strength. Bartha summarizes recent advances in the use of analogical arguments in science and provides guidelines for assessing their strengths and limitations [14]. Because of in vivo measurement limitations, a lack of detailed mechanism-based knowledge, and the fog of multi-source uncertainties, reliance on analogical reasoning and arguments is necessary to make progress in achieving a key objective, which is to begin resolving cause-effect linkages between APAP disposition and simultaneous measurements of ALT in plasma. Because of uncertainties and knowledge gaps, there is still a significant constellation of MMs having similar granularities that meet Requirements and are capable of providing equally plausible quantitative explanations of APAP-induced plasma ALT values in mice.

Consequently, establishing a reliable reverse mapping from plasma ALT values to particular mechanism features (**I** and **II**) is not yet feasible, and that reality limits the ability of ALT to serve as a mechanism-grounded biomarker of drug-induced liver injury. To improve that reality, we need to shrink the constellation of equally plausible MM-based explanations. We can accomplish that by expanding the set of validation targets to be included within future studies: add temporal measures of a small panel of other putative biomarkers [39], such as mitochondrial RNA-122, glutamate dehydrogenase, nuclear DNA fragments, APAP-protein adducts, and microRNAs. The use of APAP-protein adducts as a validation target is particularly attractive because of its direct relevance to the MM events. Doing so is also expected to diminish significantly uncertainties currently ascribed to individual variability.

The MitoD-Caused MM is a work-in-progress. It is not intended to become a finished product. For the time being, it provides a plausible biomimetic model mechanism-based explanation for how the targeted plasma ALT values are generated. The analogy is only as strong as the weakest link in the dynamic networking of MM events during an execution. New wet-lab experiments designed to challenge the MM—falsify it—are needed and will yield useful new knowledge, no matter the outcome. If the experiment falsifies a MM feature or phenomenon, one can use the results from that experiment as new validation targets and then follow the IRP to discover a set of MM improvements that enables achieving those new validation targets (while still achieving Parent MM validation targets), thus producing a more explanatory, more credible MM. If the experiment fails to falsify the targeted feature, the result strengthens the MitoD-Caused MM analogy. There may be no reason to consider such model falsification vExperiments absent the MitoD-Caused MM and the evidence supporting it. There are multiple features (among **I** and **II**) that may merit a falsification challenge. The following are three examples.

Although the magnitude of each scaling in Fig 6 differs, the temporal patterns are the same. Most of the events (**II**) driving ALT externalization are completed within the first 3 h (Fig 4 and S1 Fig). Consequently, we expect that having sequential measures of plasma ALT from each mouse within 3–6 h post-dose will be most effective in challenging those features. The results will also aid in resolving uncertainties grounded in interindividual variability. Tightly coupling wet-lab and vExperiments is scientifically sound and is an economical way to concurrently expand explanatory knowledge while chipping away at those uncertainties [8,10,12].

The amount of ALT within each vHPC that can be externalized can be another target for falsification. Absent evidence to the contrary, we specified that the amount is the same, independent of PP-to-PC location. Measures of lobular location-dependent ALT activities or ALT gene expression levels (**I**) may (or not) directly challenge that working hypothesis. Indirect evidence from experiments in rats challenges that hypothesis. Gascon-Barré et al. showed that ALT activities in PP hepatocytes are three-fold higher than those in PC hepatocytes [40]. To our knowledge, there are no reports of comparable measurements in mice.

When we Dosed the MitoD-Caused MM with virtual counterparts to 150 mg/kg and 600 mg/kg APAP doses (Fig 7), the scaled ALT-in-Mouse-Body amounts failed to mimic the mean plasma ALT values. Those failures show that the explanatory details must be different following the three doses. Results of exploratory low Dose vExperiments suggested that using a larger Leakage Threshold and an extended Death Delay range may be sufficient to mimic the referent plasma ALT values. Whereas, at the high Dose, the results of the exploratory vExperiments suggested that combining a small Leakage Threshold with extended Leakage lag time and Death Delay ranges may be sufficient. Retaining the same components and features (**I**), one can posit several scenarios for similar feature changes that may enable a single MM to achieve similarities at all three APAP doses. The following are three examples. 1) Make the parameterization of Leakage Threshold, Leakage lag time, and Death Delay ranges within each vHPC dependent on the amounts of Damage Products during the previous simulation cycle. Leakage Threshold would be inversely related to Damage Product amounts, whereas Leakage lag time and Death Delay would increase as Damage Products increase. 2) Specify independent PP-to-PC-dependent values for each of those three features, analogous to gradients used by the Parent MM (Fig 1A). Hypothesizing new PP-to-PC location-dependent features is supported indirectly by the fact that over 50% of expressed liver genes exhibit PP-to-PC expression differences [41]. 3) Specify that the Leakage threshold, Leakage Lag time, and Death Delay within a particular vHPC is influenced, via Cell-Cell communication, by the damage state of its neighbors, analogous to the communication features of the model mechanism used by Kennedy et al. [38]. However, we will need new evidence to select one scenario for further exploration.

For each of the above scenarios, the constellation of plausible yet meaningfully different biomimetic parameterizations will be significant. To improve MM-based explanatory clarity, we need finer-grain measures of release processes to serve as validation targets. With additional evidence-based constraints, in conjunction with new validation targets and strong Similarity Criteria, it is straightforward to use the Iterative Refinement Protocol to alter and add MM features and then select among competing MM-based theories. In so doing, we can shrink the plausible constellation of MMs to a manageable size [8,13,38].

## Supporting information

S1 Text There are four subsections. 1. Setting up a vExperiment at the start of an Iterative Refinement Protocol cycle. 2. Mouse components and their organization. 3. Model Mechanisms that may explain APAP-induced liver Injury. 4. Use of a Marker Compound as a Lobule-structure-Disposition interaction indicator.
(PDF)

S1 Table Description and values for important model mechanism features. A list of important configurations/parameters for model mechanistic features specifying the activity and event within vHPCs. The main features are divided into membrane transport of vCompounds, APAP binding/metabolism, damage production/amplification, damage mitigation, necrosis, and ALT externalization (the focus of the main text). In addition, the values listed correspond to the information in Fig 2 of the main text.
(DOCX)

S2 Table Values of *δ*_*i*_ for each individual mouse used in Eq 2. For the MitoD-Caused MM vExperiment, *δ*_*i*_ is the degree to which a mean ALT-in-Mouse Body amount must be skewed (amplified or diminished) to match the plasma ALT value from mouse *i*.
(PDF)

S3 Table Statistical measures of ALT in Mouse Body for MM variants. The values listed are the minimum, maximum, mean, standard deviation, variance, and coefficient of variation of the unscaled amount of ALT in Mouse Body (12 Monte Carlo trials) for the four MM variants at 3, 4.5, and 6 h post-Dose. The coefficient of variation is consistent over time and MM variants.
(PDF)

S1 Fig Temporal profiles of measurements made during executions of the parent Model Mechanism. The data in Fig 4 are from the same experiment. (A) APAP and its Metabolites, G and S, in Mouse Body. Values in B-E are centered moving averages. (B) Average amount of APAP per 1000 vHPCs within the PP, Mid-Zonal (M-Z), and PC bands identified in Fig 3C. The PP-to-PC increase in the amount of APAP per vHPC is a direct consequence of fewer PC vHPCs (Fig 3C) being exposed to the amount of incoming APAP. (C) Average amount of NAPQI within PP, M-Z, and PC bands. Differences among the PP, M-Z, and PC bands are more dramatic than those for APAP because the fraction of APAP that is Metabolized to NAPQI, rather than to G and S Metabolites, increases PP-to-PC. (D) Distance from CV of average Necrosis-Triggered events within PC and M-Z bands. The few events within the PP band are more distant. (E) Cumulative GSH depletion events. The order of the cumulative GSH Depletion profiles may seem inconsistent with the order of amounts in B and C. The explanation is that by 1 h post-Dose, the GSH Depletion Threshold for a majority of vHPCs within the PC band has been breached. However, GSH Depletion Thresholds within the M-Z band are larger, so GSH Depletion continues even though less NAPQI per APAP is being formed. (F) Cumulative Damage Mitigation events.
(TIF)

S2 Fig Measures of amounts of ALT to be released for all four MMs in Table 1. Each panel contains data from one of the three bands in Fig 3C. Within a panel, each profile is the cumulative percent of ALT that is scheduled for release at t or earlier. Once an ALT is scheduled for release, the event will occur following a Monte Carlo sampled lag time. Note the differences between the profiles for the MitoD-Caused and the nonMD Caused MMs. By 4 h post-Dose for the MitoD-Caused MM, there are no additional ALT release events are scheduled within the PC and M-Z bands. Whereas, 6 h post-Dose for the nonMD-Caused MM, ALT release events are still being scheduled within all three bands.
(TIF)

S3 Fig Dose-response relationships of Necrosis-Triggered and Necrotic events from vExperiments using the MitoD-Caused MM. The three Doses designated low, medium, and high have the wet-lab counterparts indicated in Fig 7. Average measurements are plotted at 4.5 h (A) and 12 h (B) post-Dose. Average measurements are the same at 12 h post-Dose (and 24 h post-Dose).
(TIF)
