# Supplementary material for: Contrasting model mechanisms of alanine aminotransferase (ALT) release from damaged and necrotic hepatocytes as an example of general biomarker mechanisms
Source: PLoS Comput Biol. 2020 Jun 2;16(6):e1007622. doi: 10.1371/journal.pcbi.1007622 (PMC7292418; doi:10.1371/journal.pcbi.1007622)
Supplement: S1 Text — 1. Setting up a vExperiment at the start of an Iterative Refinement Protocol cycle. 2. Mouse components and their organization. 3. Model Mechanisms that may explain APAP-induced liver Injury. 4. Use of a Marker Compound as a Lobule-structure-Disposition interaction indicator. (PDF) [file pcbi.1007622.s001.pdf]

Supporting S1 Text

1. Setting up a vExperiment at the start of an Iterative Refinement Protocol cycle

Iterative Refinement Protocol (IRP) steps are illustrated. The first 7 steps compose what is more generally known as a systems engineering process for setting and flowing requirements down to an implementation. Often, however, the very first article of a M&S project is best thought of as a "mock up," an artifact that simply provides a placeholder which steps 1-7 will evolve or replace. That placeholder also has some preliminary requirements. In our case, an arching requirement is that our models be open, mechanistic explanations. Therefore, the toolchain we start with is required to be open and, more importantly, somewhat universally extensible. For this reason, we start with a set of libraries in a general-purpose programming language on top of an open-source stack. For these models, that language is Java and the toolkit is MASON. MASON atop Java carries an implicit ontology and model structure, the most important feature of which is that it is both Discrete Event (DE) and Object-Oriented (OO).

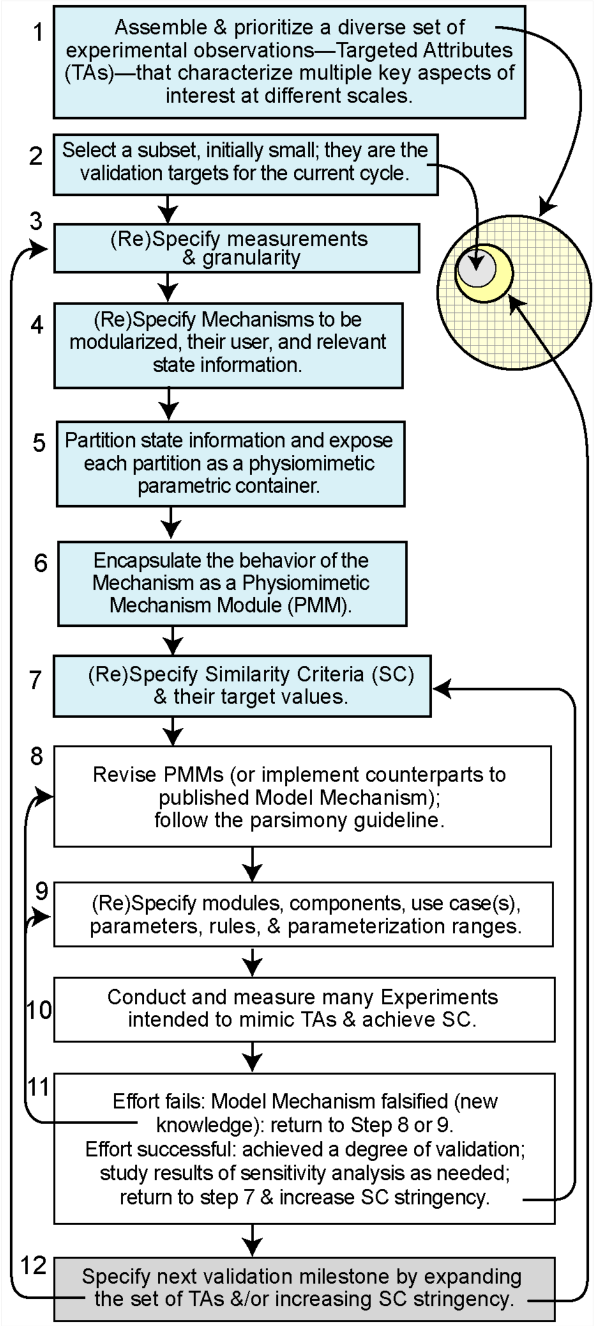

Iterative Refinement Protocol

Event ordering, seeding, explicit and implicit (pseudo) randomization, and the heap – The placeholder that launches the IRP is a DE/OO template model, where objects are scheduled for execution either with interleaved ordering or in parallel. We use pseudo-random interleaving to simulate parallelism in most cases.

Such placeholders are also structured according to the Model-View-Controller (MVC) pattern. And the measures and experimental protocols are, through steps 1-7, refined as extensions of the Viewer component in that MVC framework. The Model, Viewer, and Controller are separate and execute somewhat independently, with the Model and Viewer as slaves to the Controller. Therefore, another element of variation enters the experimental results due to the synchrony (or lack

thereof) between the execution of the Model and its measurement by the Viewer. For example, the model might execute faster than the measures, perhaps even using fractional rates. E.g. if the model executes 1.5 times faster than the measures, the measurements of a repeat experiment might show the model in different states than the first experiment. We purposefully avoid such fractional frequencies in the model presented here.

The pseudo-random numbers all descend from a single generator (Mersenne Twister) seeded with a long at the beginning. When using simulated parallelism (interleaved serialized events), this allows for near perfect repeatability. Some elements of such models are infeasible to repeat, however. For example, in MASON, a “heap” data structure is used to keep track of some events. And the Java specification allows implementations to leave such handling unspecified. This means that, in spite of using a pseudo-random algorithm, some events will be unpredictably ordered.

As steps 1-7 refine the analogy between the referent and the Model, probability distributions are programmed into the mechanism. To meet the base requirements of openness and extensibility, we use Monte Carlo (MC) sampling of those (several) pseudo-random distributions to compose a master distribution **without** having specified a “master equation” analytically. In most cases, this requires the use of uniform distributions because the presence of parameter specified distributions (e.g. Gaussian) would bias the results. However, if a particular variation in behavior of a sub-mechanism is well established in the literature, we will use that. The only non-uniform distributions in the model presented are in the length of the SSs (Gamma) and to initialize Binding capacities inside Hepatocytes (Binomial). All other distributions are uniform, specified in the parameters with a max and min.

2. Mouse components and their organization

A Mouse (Fig 1) comprises Mouse Body, vLiver, and a space to contain Dose for simulating intraperitoneal dosing. A vLiver is the number of Monte Carlo-sampled vLobule variants per experiment. One vLobule maps to a small random sample of lobular flow paths within a whole liver along with the total volume of associated tissue. It reflects the fact that in vivo APAP and other compounds entering portal vein tracts from blood get exposed to many more hepatocytes than does APAP in blood nearing the central vein. A vLobule comprises a directed graph with a particular SS object at each graph node. Flow follows the directed graph. Graph nodes are organized into three Layers, which map to conventional hepatic zones. Layer 1 = PP zone; Layer 2 = Mid-Zonal; and Layer 3 = PC zone. There are 45 nodes in Layer 1, 20 in Layer 2, and 3 in Layer 3. That structure maps directly to the quasi-polyhedral nature of hepatic lobules. At the start of each execution, all SS dimensions are Monte Carlo-sampled within constraints that enable simulating the wide variety of PP to PC flow paths that were needed to enable the same vLiver to achieve previously described pharmacokinetic Target Attributes for several different drugs [1,2].

A virtual experiment is a fixed number of Monte Carlo-sampled Mouse executions with a different pseudo-random number seed between each execution. For a 12-Monte-Carlo-execution experiment, the minimum, median, and maximum SS lengths were as follows: Layers 1: 4, 8 and 15; Layer 2: 5, 5.5 and

11; Layer 3: 8, 9 and 10; and vLobule-wide min = 17, mean = 22.5 and max = 36. Intra-Layer edges, primarily within Layer 1 (there are none in Layer 3), mimic interconnections among sinusoids. Numbers of intra- and inter-Layer edges are specified, but their node-to-node assignment are Monte Carlo-sampled for each execution.

Events occurring within a particular SS are dynamically analogous to microscopic referent events occurring within portions of sinusoids and adjacent tissue. Each SS comprises Core, BileCanal (not a factor for this work), and four same-size grids: 1) the Blood-Cell Interface (simply Interface hereafter), 2) Endothelial Cell Space, 3) Space of Disse, and 4) Hepatocyte Space. SS dimensions are Monte-Carlo specified within constraints. Endothelial Cell objects occupy 99% of the Endothelial Cell Space. They contain binders, which can bind APAP and other Solutes nonspecifically. vHPCs occupy 90% of the Hepatocyte space. An APAP object maps to a tiny fraction of an actual APAP dose. APAP Doses are ≤ 100,000 objects. Each simulation cycle (discrete time-step), a fraction of APAP in Body is transferred to PV. From there, Compounds enter Core and Interface spaces at the upstream end of all Layer 1 SSs. Extra-Cellular Compounds percolate stochastically through accessible spaces toward the CV influenced by parameter values that control local flow. Compounds that reach the distal end of Core and Interface spaces are transferred along a connecting edge to another SS. Compounds exit Layer 3 SSs into CV, where they get moved to Body. PV-to-CV gradients provide intra-Lobular location information used by each vHPC. During an execution, each simulation cycle scales to approximately 1 second.

Cells are software agents. Entry and exit of Compounds from Cells is mediated by the Cell according to the Compound's properties. Endothelial Cells contain binders that bind and release APAP (maps to non-specific binding). Binders map to a conflation of all epithelial cell components responsible for non-specific binding of the referent compound. For a typical experiment, the average number of vHPCs per vLobule was 16,165, with Layer 1 = 10,860, Layer 2 = 4,910, and Layer 3 = 720. vHPCs also contain binders. They map to a conflation of all hepatocyte components responsible for non-specific APAP binding plus all metabolic enzymes responsible for APAP metabolism along with the futile cycle in which APAP is deacetylated to *p*-aminophenol followed by rapid reacetylation back to APAP, even though the cycle is viewed as having little importance in APAP-induced hepatotoxicity in humans and mice [3].

3. Model Mechanisms that may explain APAP-induced liver Injury

Events that can occur within a vHPC each simulation cycle are illustrated in Fig 2. vHPC capabilities are identical to those used previously [4]. vHPCs contain four types of physiomimetic modules to control material entry, removal, binding and transformations: *EliminationHandler*, *MetabolismHandler*, *BindingHandler*, and *InductionHandler* (not used in this work). The order of events is (pseudo) randomized each simulation cycle.

The probability of an APAP Metabolism event and the probability that the Metabolite is NAPQI increase threefold from PV to CV. All other Metabolites are lumped together and for simplicity are divided equally between G and S, which map to the APAP glucuronide and APAP sulfate metabolites. The MM

uses vLobule-dependent values from each of five gradients [4]. Each gradient is implemented explicitly as a function of distance from PV entrance to the vHPC's position.

Each vHPC has a location-determined GSH Depletion Threshold value (Fig 2A). At early times, there is a 90% chance that as soon as a NAPQI is created, it is eliminated and the GSH Depletion Threshold counter is decremented by 1.0, which maps to depleting a fraction of a hepatocyte's available GSH. The Threshold is breached when the counter value = 0, which maps to effective GSH depletion. A small or zero Threshold value means that a vHPC is sensitive to NAPQI-caused damage. The iterative process resulting in the location-dependent GSH Depletion Threshold values was described in details by Smith et al. [4].

Each cycle following GSH depletion, there is a 50% chance that a NAPQI will be removed and replaced by (n + 1) Damage Products, one of two types. 1) A MitoD object maps to a conflation of all mitochondria-associated damage products. 2) A nonMD object maps to a conflation of all other types of damage products within a hepatocyte, including those identified with endoplasmic reticulum stress and the unfolded protein response. Each time that one NAPQI is removed, (n + 1) Damage Product objects are created. That number is needed to enable downstream events to be more fine grain, including accounting for accumulation of reactive oxygen/nitrogen species. The value of n is a pseudo-random draw from the uniform [3, 6] distribution.

When the MitoD amount > Necrosis Threshold value (which = 4 for this work), Necrosis is Triggered and the vHPC is designated Necrosis-Triggered. That transition is irreversible. When the Necrosis Threshold is breached, there is a Delay before that vHPC transitions to Necrotic. Transition to Necrotic maps to histologically distinguishable necrosis. To our knowledge, there is no current method available to measure a corresponding transition for hepatocytes in vivo. Because necrosis is a process, there is a delay between the molecular level triggering event(s) and a subsequent time when necrosis becomes clearly detectable in stained tissue sections. That internal process-delay maps the lag time in Fig 2C. There is considerable uncertainty about the timing of triggering events and histological confirmation of necrosis. In Smith et al. [4], the Death Delay lag-time is a pseudo-random draw from a uniform [Min, Max) distribution = [1.2, 12) h. However, in C57Bl/6 mice administered a 300 mg/kg APAP dose, there is little evidence of necrotic cells prior to 2 h post-dose and little evidence of further necrosis occurring after 12 h. To avoid Necrotic events prior to 2 h during this work, we set Death Delay Min = 2 h (7200 simulation cycles). For the parameterizations used, and a Dose that scales to 300 mg/kg, there are no further Necrosis-Triggered after 6 h post-Dose. We therefore set Death Delay Max = 6 h (21600 simulation cycles). Using a Death Delay distribution = [2, 6) h serves as a predicate to constrain the space searched to identify plausible MMs for ALT release.

Hepatocytes in vivo utilize multiple lobule-location-dependent mechanisms to mitigate or reverse various types of damage. We implemented a single mitigation event by repurposing a Metabolism Module. Damage Mitigation maps to a conflation of all actual mitigation/recovery processes, including processes involved in restoring homeostasis. During a Damage Mitigation event, a Damage Product (MitoD or nonMD) may be removed (Fig 2A) and a Removed object is created and added to the vHPC. The function

of Removed objects is to track Damage Mitigation events. The PV-to-CV location-specified probabilities illustrated in Fig 2A for MitoD and nonMD Damage Mitigation events were arrived at following several IRP cycles.

4. Use of a Marker Compound as a Lobule-structure-Disposition interaction indicator

It is now a standard practice to employ a Marker Compound as a type of internal standard during virtual experiments and most stages of model mechanism development, verification, and validation. Marker objects are confined to extra-Cellular spaces. Marker use in virtual experiments as analogous to use of ^{14}C-sucrose as an extracellular space marker in pharmacokinetic experiments. Marker objects are a fraction of the total Dose studied, typically 25-75%. The probability that any particular Compound (or other mobile object) will enter and exit a Cell during any time step is specified by *cellEnterExitProb* = $<p_{enter}, p_{exit}>$. For Marker, *cellEnterExitProb* = <0.0, 0.0>, and that specification is always the same.

Making code or hardware changes are analogous to changing one or more features of a wet-lab experiment protocol. There can be unintended consequences. Marker behaviors and vLiver structural features are entangled during execution. Unexpected changes in regularly measured Marker behaviors during executions provide evidence of an unintended consequence of either a software change or a Compound-Compound interaction. An example is the occurrence of a non-biomimetic, Dose-dependent bottleneck as Compounds as they transition from one space to another. Because Marker behavior, vLiver structure and vLobular spaces are entangled during execution, for any change in Marker behavior, we will likely observe some degree of change in the behavior of the Compound(s), such as APAP and ALT, on which the experiment is focused.

There are two additional benefits of Marker use: measurements of Marker can demonstrate a minimal conservation of mass, and Marker behaviors provide a quantitative link to results of experiments from all previous versions of vMouse and vLiver that employed Marker use.

All data and the code are available here: https://simtk.org/projects/aili and https://simtk.org/home/isl/.

References

1. Yan L, Ropella GEP, Park S, Roberts MS, Hunt CA. Modeling and simulation of hepatic drug disposition using a physiologically based, multi-agent in silico liver. Pharm Res. 2008; 25(5):1023-1036. doi: 10.1007/s11095-007-9494-y PMID: 18044012

2. Yan L, Sheihk-Bahaei S, Park S, Ropella GEP, Hunt CA. Predictions of hepatic disposition properties using a mechanistically realistic, physiologically based model. Drug Metab Dispos. 2008; 36(4):759-768. doi: 10.1124/dmd.107.019067 PMID: 18227144

3. Miyakawa K, Albee R, Letzig LG, Lehner AF, Scott MA, Buchweitz JP, et al. Cytochrome P450-independent mechanism of acetaminophen-induced injury in cultured mouse hepatocytes. J Pharmacol Exp Ther. 2015; 354(2):230-237. doi: 10.1124/jpet.115.223537 PMID: 26065700

4. Smith AK, Petersen BK, Ropella GEP, Kennedy RC, Kaplowitz N, Ookhtens M, et al. Competing mechanistic hypotheses of acetaminophen-induced hepatotoxicity challenged by virtual experiments. PLoS Comput Biol. 2016; 12(12):e1005253. doi: 10.1371/journal.pcbi.1005253 PMID: 27984590
