# Supplementary material for: Contrasting model mechanisms of alanine aminotransferase (ALT) release from damaged and necrotic hepatocytes as an example of general biomarker mechanisms
Source: PLoS Comput Biol. 2020 Jun 2;16(6):e1007622. doi: 10.1371/journal.pcbi.1007622 (PMC7292418; doi:10.1371/journal.pcbi.1007622)
Supplement: S1 Table — A list of important configurations/parameters for model mechanistic features specifying the activity and event within vHPCs. The main features are divided into membrane transport of vCompounds, APAP binding/metabolism, damage production/amplification, damage mitigation, necrosis, and ALT externalization (the focus of the main text). In addition, the values listed correspond to the information in Fig 2 of the main text. (DOCX) [file pcbi.1007622.s002.docx]

**S1 Table.**

| **Model Mechanistic Features (events & activities)** | **MitoD-Model** |
| --- | --- |
| membrane crossing |  |
| APAP/PAP | TRUE |
| G/S | TRUE |
| N | FALSE |
| nonMD/MitoD | FALSE |
| Repair | FALSE |
| ALT | FALSE |
| PAP cycle | Not applicable |
| APAP binding | *bindProb* = 0.1, *bindCycles* = 10 |
| APAP metabolism | *rxnProb*<PV,CV> = <0.5,0.99> |
| G/S | *prodProb*<PV,CV> = <0.33,0.05> |
| N | *prodProb*<PV,CV> = <0.34,0.9> |
| GSH depletion | *gshDepletionRange*<PV,CV> = <8.0,0.0> |
| Damage production from N |  |
| N binding | *bindProb* = 0.5, bindCycles = 10 |
| nonMD/MitoD | *rxnProb*<PV,CV> = <0.5,0.5> |
| nonMD/MitoD | *prodProb*<PV,CV> = <0.5,0.5> |
| Damage amplification |  |
| nonMD/MitoD | *AmpRange*<uniform> = <3,6> |
| Damage mitigation |  |
| nonMD |  |
| binding | *bindProb* = 0.99, bindCycles = 10 |
| reaction | rxnProb<PV,CV> = <0.2,0.8> |
| gradient | linear |
| Repair production | *prodProb*<PV,CV> = <1,1> |
| MitoD |  |
| binding | *bindProb* = 0.99, *bindCycles* = 10 |
| reaction | *rxnProb*<PV,CV> = <0.9,0.0> |
| gradient | sigmoid |
| Repair production | *prodProb*<PV,CV> = <1,1> |
| Necrosis |  |
| MitoD threshold | *necrosisRange*<PV,CV> = <4, 4> |
| Time delay | *necrosisDelay*<Min,Max> = <7200,21600> |
| ALT release |  |
| Amount/vHPC | *ALTamount* = 5 |
| Leakage Threshold | *ALTthreshold* = 1 |
| Lag delay | *transportDelay*<Min,Max> = <2700,18000> |
