# Supplementary material for: Contrasting model mechanisms of alanine aminotransferase (ALT) release from damaged and necrotic hepatocytes as an example of general biomarker mechanisms
Source: PLoS Comput Biol. 2020 Jun 2;16(6):e1007622. doi: 10.1371/journal.pcbi.1007622 (PMC7292418; doi:10.1371/journal.pcbi.1007622)
Supplement: S2 Table — For the MitoD-Caused MM vExperiment, δi is the degree to which a mean ALT-in-Mouse Body amount must be skewed (amplified or diminished) to match the plasma ALT value from mouse i. (PDF) [file pcbi.1007622.s003.pdf]

S2 Table.

Mouse	δ	Mouse	δ	Mouse	δ
1	1.46	7	1.31	13	1.17
2	0.96	8	1.29	14	1.09
3	1.31[1]	9	1.18	15	0.98
4	1.15[1]	10	1.11	16	0.84
5	0.84[1]	11	0.92	17	0.61
6	0.35[1]	12	0.87	18	0.43

[1] These values resulted from the MitoD-Caused$_{exLT}$ MM experiment.
