# Supplementary material for: Contrasting model mechanisms of alanine aminotransferase (ALT) release from damaged and necrotic hepatocytes as an example of general biomarker mechanisms
Source: PLoS Comput Biol. 2020 Jun 2;16(6):e1007622. doi: 10.1371/journal.pcbi.1007622 (PMC7292418; doi:10.1371/journal.pcbi.1007622)
Supplement: S3 Table — The values listed are the minimum, maximum, mean, standard deviation, variance, and coefficient of variation of the unscaled amount of ALT in Mouse Body (12 Monte Carlo trials) for the four MM variants at 3, 4.5, and 6 h post-Dose. The coefficient of variation is consistent over time and MM variants. (PDF) [file pcbi.1007622.s004.pdf]

S3 Table.

	Necrotic-only	Dual-Cause	nonMD-Caused	MitoD-Caused
**@ 3 h**				
min	263	2654	1889	1909
max	450	2960	2210	2234
mean	347.17	2824.33	2080.11	2048.08
std. dev.	48	101.06	85.9	95.31
variance	2304	10213.12	7378.81	9084
coeff. var.	0.14	0.04	0.04	0.05
**@ 4.5 h**				
min	1839	5558	4519	3987
max	2152	6153	4959	4700
mean	1993.42	5872.33	4739.83	4329.83
std. dev.	97.63	176.55	122.59	174.48
variance	9531.62	31169.90	15028.31	30443.27
coeff. var.	0.05	0.03	0.03	0.04
**@ 6 h**				
min	3931	7613	6773	5650
max	4391	8518	7259	6525
mean	4062.83	8085	7018.44	6047.5
std. dev.	130.12	254.8	157.85	215.14
variance	16931.21	64923.04	24916.62	46285.22
coeff. var.	0.03	0.03	0.02	0.04
