# Supplementary figures and images for: Contrasting model mechanisms of alanine aminotransferase (ALT) release from damaged and necrotic hepatocytes as an example of general biomarker mechanisms
Source: PLoS Comput Biol. 2020 Jun 2;16(6):e1007622. doi: 10.1371/journal.pcbi.1007622 (PMC7292418; doi:10.1371/journal.pcbi.1007622)

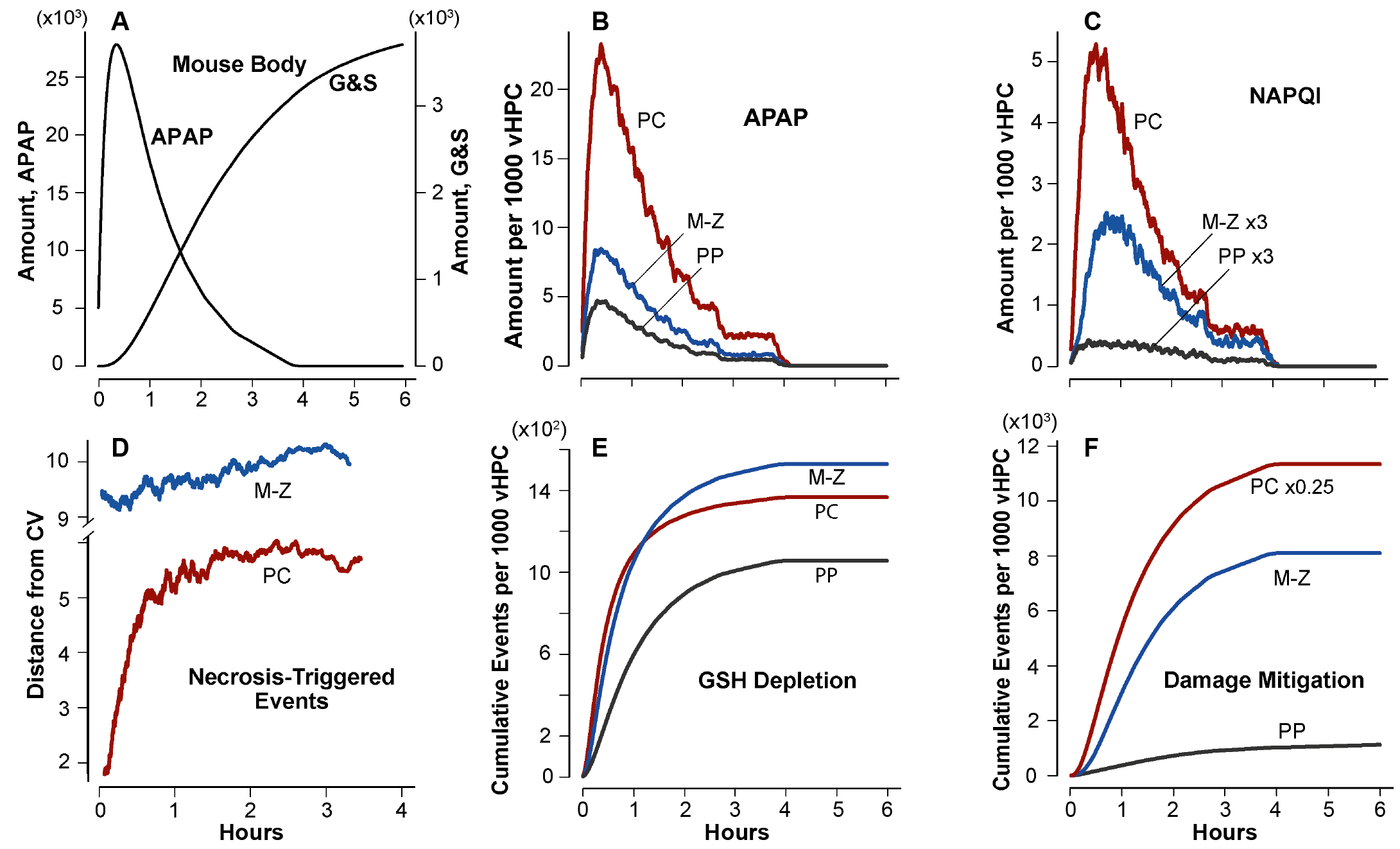

Supplement: S1 Fig — The data in Fig 4 are from the same experiment. (A) APAP and its Metabolites, G and S, in Mouse Body. Values in B-E are centered moving averages. (B) Average amount of APAP per 1000 vHPCs within the PP, Mid-Zonal (M-Z), and PC bands identified in Fig 3C. The PP-to-PC increase in the amount of APAP per vHPC is a direct consequence of fewer PC vHPCs (Fig 3C) being exposed to the amount of incoming APAP. (C) Average amount of NAPQI within PP, M-Z, and PC bands. Differences among the PP, M-Z, and PC bands are more dramatic than those for APAP because the fraction of APAP that is Metabolized to NAPQI, rather than to G and S Metabolites, increases PP-to-PC. (D) Distance from CV of average Necrosis-Triggered events within PC and M-Z bands. The few events within the PP band are more distant. (E) Cumulative GSH depletion events. The order of the cumulative GSH Depletion profiles may seem inconsistent with the order of amounts in B and C. The explanation is that by 1 h post-Dose, the GSH Depletion Threshold for a majority of vHPCs within the PC band has been breached. However, GSH Depletion Thresholds within the M-Z band are larger, so GSH Depletion continues even though less NAPQI per APAP is being formed. (F) Cumulative Damage Mitigation events. (TIF) [file pcbi.1007622.s005.tif]

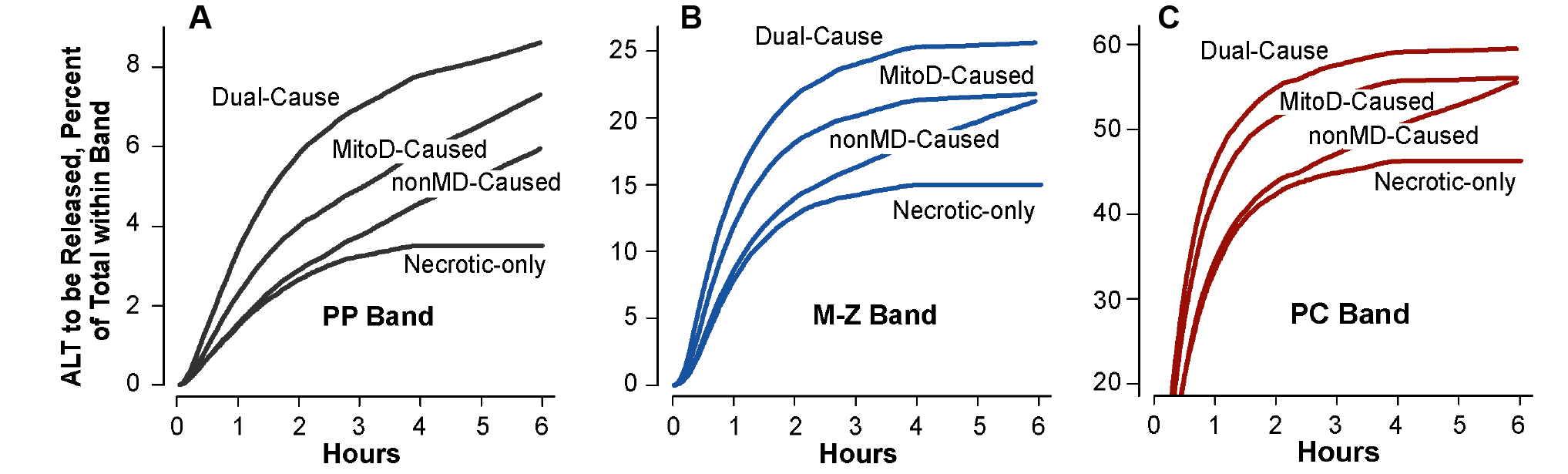

Supplement: S2 Fig — Each panel contains data from one of the three bands in Fig 3C. Within a panel, each profile is the cumulative percent of ALT that is scheduled for release at t or earlier. Once an ALT is scheduled for release, the event will occur following a Monte Carlo sampled lag time. Note the differences between the profiles for the MitoD-Caused and the nonMD Caused MMs. By 4 h post-Dose for the MitoD-Caused MM, there are no additional ALT release events are scheduled within the PC and M-Z bands. Whereas, 6 h post-Dose for the nonMD-Caused MM, ALT release events are still being scheduled within all three bands. (TIF) [file pcbi.1007622.s006.tif]

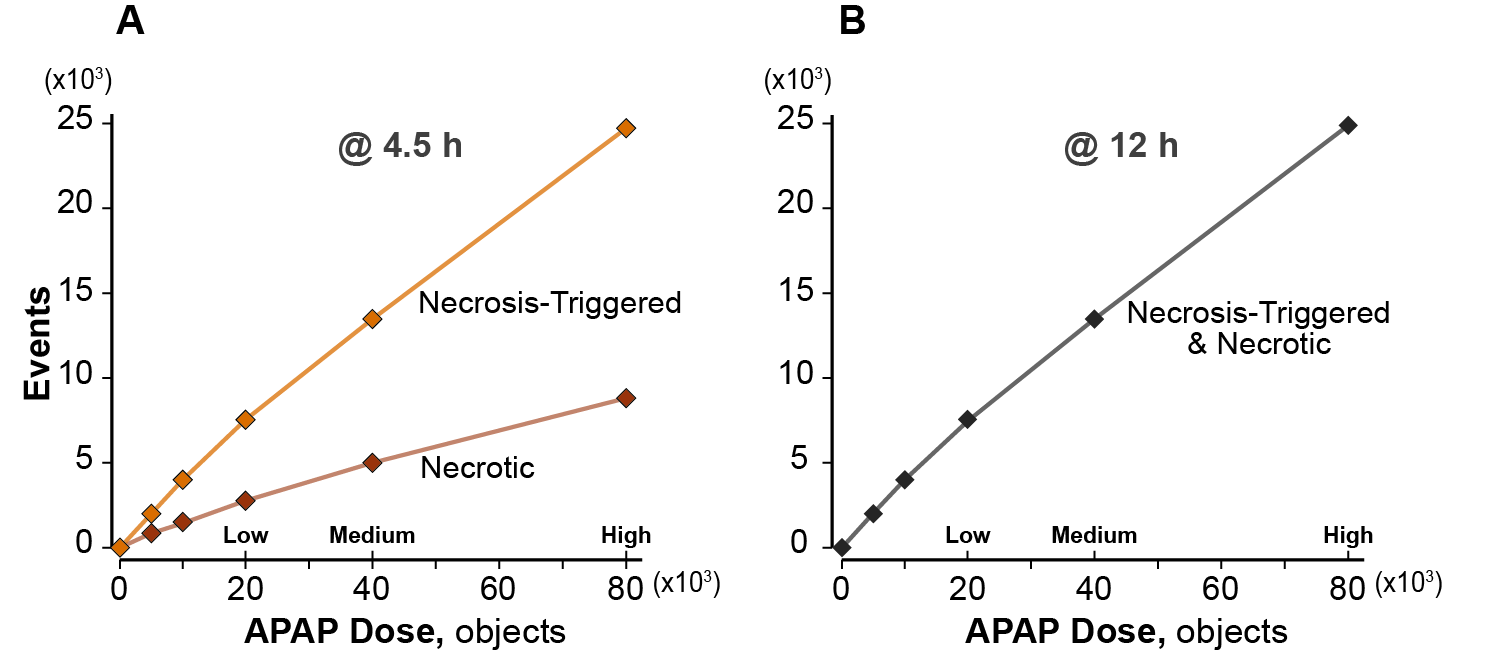

Supplement: S3 Fig — The three Doses designated low, medium, and high have the wet-lab counterparts indicated in Fig 7. Average measurements are plotted at 4.5 h (A) and 12 h (B) post-Dose. Average measurements are the same at 12 h post-Dose (and 24 h post-Dose). (TIF) [file pcbi.1007622.s007.tif]
